# Dynamic mechanisms for membrane skeleton transitions

**DOI:** 10.1242/jcs.263473

**Published:** 2025-02-28

**Authors:** Mayte Bonilla-Quintana, Andrea Ghisleni, Nils C. Gauthier, Padmini Rangamani

**Affiliations:** ^1^ Department of Mechanical and Aerospace Engineering, University of California San Diego, La Jolla, CA 92093, USA; ^2^ Institute FIRC of Molecular Oncology (IFOM), Via Adamello 16, 20139 Milan, Italy; ^3^ Department of Pharmacology, School of Medicine, University of California San Diego, La Jolla, CA 92093, USA

**Keywords:** Spectrin, Actomyosin, Cell mechanics, Cytoskeleton

## Abstract

The plasma membrane and the underlying skeleton form a protective barrier for eukaryotic cells. The molecular players forming this complex composite material constantly rearrange under mechanical stress. One of those molecules, spectrin, is ubiquitous in the membrane skeleton and linked by short actin filaments. In this work, we developed a generalized network model for the membrane skeleton integrating myosin contractility and membrane mechanics to investigate the response of the spectrin meshwork to mechanical loading. We observed that the force generated by membrane bending is important in maintaining a regular skeletal structure, suggesting that the membrane is not just supported by the skeleton, but actively contributes towards the stability of the cell structure. We found that spectrin and myosin turnover are necessary for the transition between stress and rest states in the skeleton. Simulations of a fully connected network representing a whole cell show that the surface area constraint of the plasma membrane and volume restriction of the cytoplasm enhance the stability of the membrane skeleton. Furthermore, we showed that cell attachment through adhesions promotes cell shape stabilization.

## Teaser

**Summary:** A model of the spectrin skeleton reveals mechanisms that are necessary to respond to external stresses. Moreover, it shows how global signals support skeletal structure and enhance its stability.

## INTRODUCTION

To accomplish some of their primary functions, such as motility and cell division, eukaryotic cells need to endure many mechanical challenges (Leterrier and Pullarkat, 2022). For example, axons extend long distances and can experience an increase in tension during mechanical deformation. A specific case is the stretch of the sciatic nerves when the ankle flexes due to the specific positioning of the joints (Andrade et al., 2016). During normal extension and flexion of the joints, the sciatic nerve has a 5- to 10-fold increase in the strain near the joints (Phillips et al., 2004). At the other length scale, red blood cells (RBCs), roughly 8 μm in diameter, deform to go through capillaries and the amount of deformation depends on the shear stress they experience (Hochmuth and Mohandas, 1972). The ability of these cells to resist a wide range of deformations is due to the load-bearing features of their structure. Broadly, cell architecture is determined by the canonical cytoskeleton and the membrane skeleton (Li et al., 2023). The former is a three-dimensional (3D) network of filaments, such as actin filaments and microtubules, which provide support to organelles and change their configuration to allow different cell functions. The membrane skeleton consists of a spectrin network beneath the plasma membrane.

**Fig. 1.**
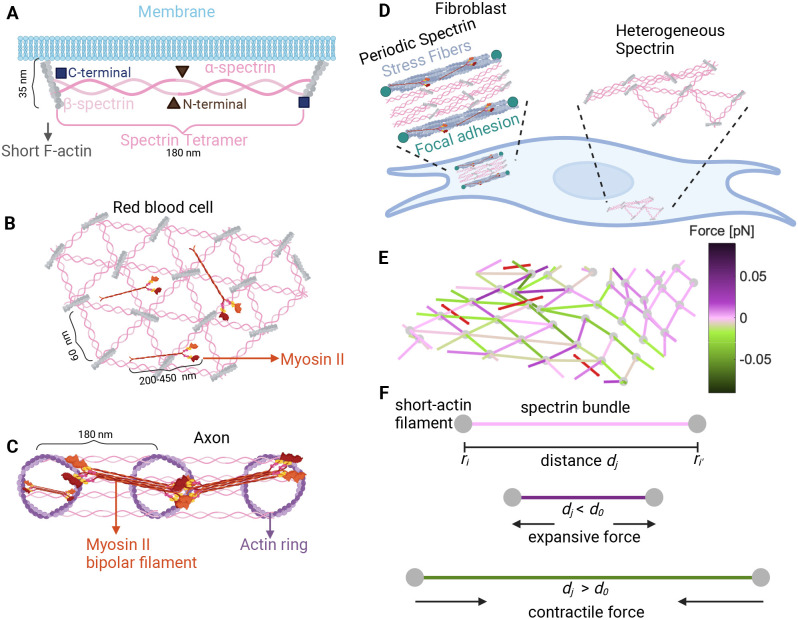
**Different configurations of the membrane skeleton.** (A) A spectrin tetramer spanning between short actin filaments. (B) The hexagonal actin–spectrin meshwork configuration in red blood cells. Myosin generates contractility that might preserve the cell shape (Smith et al., 2018). (C) Periodic actin–spectrin meshwork configuration in axons. Myosin heavy chains crosslink adjacent actin rings, likely providing tension. Myosin might also span individual rings, providing contraction (Costa and Sousa, 2020). (D) In fibroblasts, the actin–spectrin meshwork has a heterogeneous and dynamic configuration (Ghisleni et al., 2024). (E) Schematic of the simulated 3D network model. The red lines correspond to myosin, gray nodes to short F-actin, and edges to spectrin, color coded for the force generated by the spring element. (F) Schematic representation of the forces generated by the spectrin edges when their length (*d_j_*) differs from the resting length (*d*_0_).

Spectrins are proteins that form scaffolds with other molecules inside the cell and confer rapid solid-like shear elasticity to support in-plane shear deformations (Leterrier and Pullarkat, 2022; Nans et al., 2011). The spectrin scaffold is constructed by attaching the ends of the spectrin rod-like heterotetramers to junctional complexes composed of short F-actin and other proteins (Nans et al., 2011), forming an actin–spectrin meshwork (Fig. 1A). These junctional complexes are some of the structures that connect the spectrin scaffold to the plasma membrane. Although cytoskeletal molecules such as actin and microtubules use active polymerization to support mechanical loading on cells, spectrin accomplishes its role either by dynamically unfolding or by disassembling the dimer–dimer links (Leterrier and Pullarkat, 2022; An et al., 2002; Rief et al., 1999). When a spectrin tetramer is pulled, its repeats unfold and can exhibit a 2.6-fold increase in contour length. The unfolding of the repeats depends on the force and velocity of the pulling (Leterrier and Pullarkat, 2022; Rief et al., 1999). The structural organization of the spectrin scaffold depends on the cell type (Baines, 2009) and, as shown more recently, on subcellular location (Ghisleni et al., 2020, 2024). In RBCs, the two main paralogs of spectrin, αI (encoded by *SPTA1*) and βI (encoded by *SPTB*), associate laterally and in an antiparallel manner to form long and flexible heterodimers (Nans et al., 2011; Teliska and Rasband, 2021). Interactions between the N-terminus of α-spectrin and the C-terminus of β-spectrin produce bipolar heterotetramers (Nans et al., 2011) (Fig. 1A). The junctional complexes form a pseudo-hexagonal lattice (Nans et al., 2011), which was thought to be regular (Fig. 1B). However, recent experiments showed that F-actin in junctional complexes forms irregular, non-random clusters (Nowak et al., 2022).

The configuration of the spectrin scaffold in neurons differs in the soma, axon and dendrites even though it is formed by the same elements: spectrin, actin and myosin. In axons, α- and β-spectrin link evenly distributed actin rings, thereby regulating the spacing between rings (∼180–190 nm) and giving mechanical support to the membrane (Xu et al., 2013; Costa and Sousa, 2020) (Fig. 1C). A similar periodic skeleton configuration was found in dendrites (D'Este et al., 2015), but the configuration in the soma is similar to that in RBCs (Han et al., 2017). In fibroblasts (Fig. 1D), spectrin is spatially distributed in regions where the cell edge retracts and there is a low density of actin (Ghisleni et al., 2020). Ghisleni et al. (2020) showed that the distribution of spectrin is dynamic and it changes during mechanical challenges such as cell adhesion, contraction, compression, stretch and osmolarity changes. Moreover, recent studies from our group revealed that βII-spectrin (encoded by *SPTBN1*) in fibroblasts transitions between a heterogeneous configuration resembling that in RBCs to a periodic configuration resembling that in axons (Ghisleni et al., 2024). Such a transition is driven by actomyosin contractility.

Previously, we used a theoretical model to show that the experimentally observed actin–spectrin transitions in fibroblasts require spectrin detachment from the short F-actin (Ghisleni et al., 2024). Interestingly, some experimental evidence suggests that the actin–spectrin meshwork in RBCs (An et al., 2002; Nowak et al., 2022; Smith et al., 2018; Turlier et al., 2016; Park et al., 2010; Rodríguez-García et al., 2015) and axons (Wang et al., 2020) is also dynamic. In this work, we sought to understand how a minimal system of short actin filaments, myosin motors and spectrin tetramers can give rise to a wide range of network configurations and confer mechanoprotective capabilities in the cellular context. We used a network model of springs and cables to represent the membrane skeleton (Vignaud et al., 2021; Guthardt Torres et al., 2012; Paul et al., 2008; Bischofs et al., 2008; Ghisleni et al., 2024) (Fig. 1E,F) and incorporated the response of the membrane to mechanical stress (Li et al., 2005). Many models focus on the molecular details of spectrin (Jäger et al., 2022; Li et al., 2005; Peng et al., 2013; Gov and Safran, 2005; Li and Lykotrafitis, 2014). However, in this work, we focused on the mechanical interactions and the resulting shape deformations at larger scales, rather than on the molecular details. This approach allowed us to examine the interaction between spectrin and the continuous turnover of myosin in a whole cell. Because this interaction between spectrin and myosin is found in many types of cells (Xu et al., 2013; Costa and Sousa, 2020; Ghisleni et al., 2020, 2024; Nowak et al., 2022), our model aims to give a general description and its findings are not exclusive to a certain cell type.

Using this model, we sought to answer the following questions: how does membrane bending interact with the actin–spectrin meshwork? How do myosin contraction and its stochastic addition and removal alter the meshwork? Which mechanisms balance the stochastic action of myosin on a whole cell to achieve shape stability? Finally, how do adhered versus detached cells adjust their actin–spectrin meshwork dynamics to conserve their shape? We observed that the balance between the force generated by the bending energy of the membrane and the force generated by spectrin lowers the stress in the membrane. This finding suggests a feedback mechanism between the skeleton and the membrane instead of just the accepted function of the skeleton in providing mechanical support to the membrane. We found that without spectrin unbinding and rebinding to junctional complexes and the action of myosin contraction and its stochastic addition and removal, the actin–spectrin meshwork remains clustered after contractile stress is removed. Therefore, these features of spectrin and myosin are necessary for recovering the pre-stressed configuration of the membrane skeleton. Moreover, our model predicts an optimal number of myosin rods for skeleton recovery from the imposed stress. We showed that, although the membrane skeleton is dynamic, it can maintain cell shape when no stress is induced by considering global membrane signals, such as surface area constraint of the plasma membrane and volume restriction of the cytoplasm. We also found that the interplay between the membrane skeleton and the substrate attachments can render stability to adhered cells. We anticipate that our model predictions have implications for a wide range of mechanoprotective scenarios in which the spectrin meshwork plays a critical role.

## RESULTS

### Qualitative description of the model

We propose a general 3D mesoscopic model for the membrane skeleton to examine its changes in morphology and mechanical properties. This model builds on the two-dimensional (2D) model presented in Ghisleni et al. (2024). The basic components of the model are an actin–spectrin meshwork (module 1) attached to the extracellular matrix (ECM) through connectors (module 2), which can induce stress and result in a change in the meshwork configuration. The forces generated by the membrane (module 3) and myosin (module 4) also affect the evolution of the meshwork configuration. Thus, a balance between the forces generated by the actin–spectrin meshwork, the membrane, myosin and connectors dictates the evolution of the meshwork configuration (module 5). Following Ghisleni et al. (2024), instead of focusing on specific values for the different model parameters, which are difficult to obtain experimentally and can diverge for different types of cells, we used values that allow us to qualitatively represent the meshwork dynamics. Thus, unlike previous modeling efforts that only studied one type of cell (Jäger et al., 2022; Li et al., 2005, 2007, 2018; Peng et al., 2013; Dubey et al., 2020; Zhang et al., 2017; Chai et al., 2023), our model is general. The model parameters are provided in Table 1.

**Table 1.** Model parameters

Symbol	Definition	Units	Value	Reference
*ζ*	Drag coefficient	pN s/nm	1.25	Ghisleni et al. (2024)
Δ_*t*_	Time step length	s	0.002	Ghisleni et al. (2024)
**Spectrin**				
*k*_*s*,*S*_	Spring constant	pN/nm	1	Ghisleni et al. (2024)
*d*_0,*S*_	Resting length	nm	180	Ghisleni et al. (2024)
*F* ^*th*^	Force threshold for detachment	pN	0.05	Ghisleni et al. (2024)
**Connecting edges**				
*k*_*s*,*C*_	Spring constant	pN/nm	1	Fitted
*d*_0,*C*1_	Resting length for compression	nm	450	Fitted
*d*_0,*C*2_	Resting length for extension	nm	270	Fitted
*d*_0,*C*3_	Resting length for shear	nm	270	Fitted
*d*_0,*C*4_	Resting length for adhered cell	nm	75	Fitted
*d*_*I*,*C*1_	Initial length for isotropic stress	nm	360.1389	Fitted
*d*_*I*,*C*3_	Initial length for shear	nm	1138.4638	Fitted
*d*_*I*,*C*4_	Initial length for adhered cell	nm	[100,163.05]	Fitted
**Membrane**				
*k*_*b*_	Bending constant	pN nm	820 (200*k*_*B*_*T*)	Li et al. (2005)
*θ*_0_	Spontaneous curvature angle	°	0	Fitted
*k*_*A*_	Area constant	pN nm	0.0380 [300 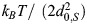 ]	Based on Li et al. (2005)
**Myosin**				
*k*_*c*,*M*_	Cable constant	pN/nm	0.1071	Ghisleni et al. (2024)
*ϕ*_*a*_	Myosin addition rate	1/s	0.01	Ghisleni et al. (2024)
*ϕ*_*r*_	Myosin removal rate	1/s	0.0063	Ghisleni et al. (2024)
*d*_*min*_	Minimum length	nm	135	Ghisleni et al. (2024)
*d*_*max*_	Maximum length	nm	450	Ghisleni et al. (2024)

As the aim of our model is to examine the membrane skeleton response to applied stresses and the mechanisms for cell shape stability, we represented the skeleton as a network of springs. Although this representation lacks molecular details at the nanoscale, it is well suited to investigate mechanical contributions and perform simulations of the whole cell. Moreover, this representation is general to different types of cells. Because our focus is on mechanical responses, we do not explicitly model ATP-driven processes that could be involved (Rodríguez-García et al., 2015; Park et al., 2010; Turlier et al., 2016).

#### Module 1 – mechanics of the actin–spectrin meshwork

The actin–spectrin meshwork comprises *N_e_* edges connected by *N_n_* nodes, representing spectrin bundles and short F-actin, respectively (Fig. 1E,F). The position of each node, *i*, is given by 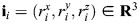, where *i*∈{1,…,*N*_*n*_}. Hereon, vector quantities are represented using bold letters. The spectrin edges behave like springs with potential *U^spring,S^*, given by:
(1)

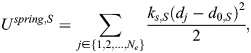
where *d_j_* is the edge length, *d*_0,*S*_ is the resting length and *k_s_*_,*S*_ is the spring stiffness. The edge *j* spans between the node *i* and *i*ʹ and has a length equal to 
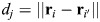
. The force generated by *U*^*spring*,*S*^ is:
(2)

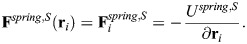
Fig. 1F shows the force generated by the spring elements when the length *d_j_* differs from the resting length *d*_0,*S*_: if the edge length is smaller than the resting length, an expansive force is generated, and if the length is larger than the resting length, a contractile force is generated. If the edge length is equal to the resting length, the nodes, which represent actin short filaments, will remain in the same position. See module 5 for details on the evolution of the position of actin nodes.

##### Spectrin unbinding and rebinding

Spectrin dissociates the dimer–dimer links by proteolytic cleavage (An et al., 2002). We included the spectrin–spectrin dissociation mechanism in our model by removing the spectrin edges that generate an expanding force greater than a threshold force (*F^th^*), i.e. 
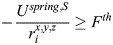
, as in Ghisleni et al. (2024). We also modeled the rebinding of the unbound spectrin edges to promote network recovery. Although different rules for spectrin rebinding can be applied, we chose the simplest case, assuming that spectrin tetramers dissociate into dimers at the N-terminal region. Hence, we expect spectrin dimers not to drift away from their current location and be more likely to connect with their previous pair to form tetramers. Moreover, this rule guarantees lower expanding force in the recently connected spectrin edges. Thus, we allowed the unbound spectrin edges to rebind when the distance between the two actin nodes to which an edge was connected equaled the resting length (*d*_0,*S*_).

#### Module 2 – induced stress by connection to focal adhesions

To induce stress on the actin–spectrin meshwork, we introduced a new type of spring edge that connects the periphery of the meshwork with fixed nodes representing focal adhesions in the extracellular space (Fig. S1A, black lines and circles). These connector edges have spring constant *k*_*s*,*C*_ and resting length *d*_0,*C*_. We assumed that the focal adhesions are 10 nm lower than the spectrin network in the *z*-axis, which accounts for the membrane thickness (4–10 nm; Daintith, 2009; Kuchel and Ralston, 1988). The initial height difference between the actin and focal adhesion nodes establishes a 3D configuration in the meshwork. The connector edges are attached to spectrin edges through protein complexes, instead of short actin filaments. Although the protein complex nodes update their position as described in module 5, we did not consider these nodes for the membrane force calculations (module 3).

#### Module 3 – membrane forces

##### Bending energy

To model the energy generated by the membrane bending (*E_b_*), we followed Li et al. (2005) and assumed that the effects of the lipid bilayer on the cytoskeleton are transmitted via transmembrane proteins and can be represented by coarse-grained local free energies. Therefore, the bending energy of the membrane affects the short F-actin nodes that are anchored to the membrane. This assumption allows us to use the actin–spectrin meshwork to calculate the bending energy as:
(3)

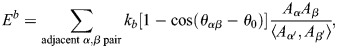
where 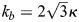, *κ* is the average bending modulus of the lipid membrane (Boal and Rao, 1992), and *θ*_0_ is the spontaneous curvature angle between two adjacent triangles *α* and *β* formed by spectrin bundles (Fig. S1B). As in Li et al. (2005), cos(*θ*_*αβ*_−*θ*_0_)=cos*θ*_*αβ*_cos*θ*_0_+sin*θ*_*αβ*_sin*θ*_0_, where 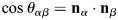 and 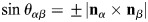. Here, sin*θ*_*αβ*_ is positive if 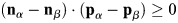. The vectors **n** and **p** represent the normal that points to the exterior and the position of the center of the triangle, respectively (Fig. S1B). Note that in the simulation, the spectrin meshwork can be irregular. Therefore, the contribution of two adjacent triangles is weighted by their area product *A*_*α*_*A*_*β*_ and normalized by the mean product over all the triangle pairs 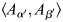 (Li et al., 2005). Hence, smaller pairs of triangles have less contribution to the bending energy. *E*^*b*^ generates a force 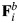, given by:
(4)

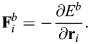
We have neglected the anchorage of spectrin to the plasma membrane through ankyrin for calculating the bending energy. We omitted ankyrin in the model because it binds to the middle of the spectrin tetramer and the model only represents full spectrin tetramers as edges. Previous work modeling a mid-point attachment (ankyrin) on the edges corresponding to spectrin bundles, which are represented as two springs in series, showed that the network stiffness can lower when the attachments are removed (Gov, 2007). We assumed that this effect in the network is comparable to removing the spectrin edges in our model. Thus, considering only short F-actin anchorage at the end of spectrin is sufficient for our simplified representation of spectrin tetramers. Moreover, the function of the spectrin–ankyrin assembly is mostly associated with the organization of membrane proteins in domains (Bennett and Healy, 2008). Hence, we do not expect changes in membrane bending.

##### Surface area constraint of the plasma membrane

We assumed that the membrane surface area is conserved in the region of interest and added a surface area constraint. This constraint generates a force:
(5)

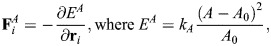
with initial surface area *A*_0_ and area constant *k*_*A*_. Hence, the total force generated by the membrane (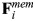) is given by:
(6)

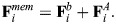


##### Volume exclusion of the cytoplasm

When simulating closed geometries to mimic cells, we assumed that cells do not shrink indefinitely and implemented volume exclusion in the model that accounts for the organelles and contents of the cytosol. To do this, we restricted the movement of F-actin nodes to a volume 15% smaller than the initial volume. If the F-actin node enters this restricted volume, it is reset to its previous value.

Note that our surface area constraint and volume exclusion descriptions do not include the molecular details involved in these processes. We made this simplifying assumption to reduce the complexity of the model. Moreover, mechanisms that regulate membrane surface area and volume have opposite effects (Gauthier et al., 2012). For example, in the membrane surface area regulation by membrane trafficking, an increase in membrane tension promotes exocytosis, which drives membrane area increase. Consequently, membrane tension drops, which then induces activation of endocytosis, leading to a reduction in surface area and a potential increase in tension. In our model, the surface area constraint of the plasma membrane and the volume exclusion of the cytoplasm serve as global signals, indicating whether the cell surface area or volume has changed from its steady-state value and inducing changes to return to it.

#### Module 4 – myosin dynamics

We followed Ghisleni et al. (2024) and added myosin as edges with cable potential energy *U^cable^*^,*M*^, where:
(7)

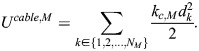
Here, *k*_*c*,*M*_ is the tensile force applied by myosin motors, *d_k_* is the edge length and *N*_*M*_ is the number of myosin edges, which are attached to the center of the triangles formed by spectrin edges (Fig. 1C). We assumed that the force generated by the myosin edges 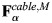 is equally distributed among the three actin nodes joining the triangle formed by the spectrin edges. Thus, the force generated by myosin edges in the F-actin nodes is given by:
(8)

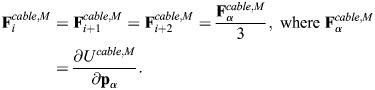


Here, **p**_α_ is the location of the center of the triangle (Fig. S1B).

Note that the cable elements only generate a contractile force. If one of the edges of the spectrin triangle is unbound, then the myosin edge tries to attach to a nearby triangle within a distance of *d*_*max*_ (Fig. S1C). If there are no triangles nearby, the myosin edge is removed from the simulation. We chose this rule because it avoids a sudden change in the position of the actin nodes attached to the missing spectrin edge and allows dynamic changes of myosin position resembling the pulsative behavior seen in experiments (Ghisleni et al., 2024). However, myosin contractility at the membrane is possible without a spectrin network. A myosin edge is also removed from the simulation if its length is less than *d*_*min*_. Note that if myosin is allowed to shrink indefinitely (*d*→0), then its force grows in an unbounded manner 
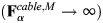
. Moreover, removing myosin prevents its concentration over time, in line with experimental observations of short-lived myosin pulses as shown in Ghisleni et al. (2024).

##### Stochastic addition and removal of myosin edges

Myosin edges are added to and removed from the network randomly at the rates *ϕ*_*a*_ and *ϕ*_*r*_, respectively.

#### Module 5 – evolution of the actin–spectrin meshwork

When stresses are induced to the actin–spectrin meshwork, the actin nodes move to restore the mechanical equilibrium, given by:
(9)

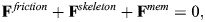
where
(10)

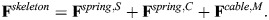
**F***^skeleton^* is the force generated by the different elements describing the dynamics of the skeleton and 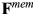 is the force generated by the membrane (Eqn 6). Note that 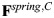 and 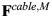 act as an external load to the actin–spectrin meshwork, driving it away from equilibrium while 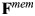 counteracts shape deformations.

Cells are surrounded by other cells and the ECM. Therefore, in Eqn 9, the forces generated by the membrane and skeleton are balanced by a friction force (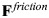). This friction force is created by the viscous dissipation between the movement of the actin nodes and the cell anchorage points to the ECM (focal adhesions) or other cells (cell junctions). Therefore,
(11)

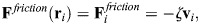
where *ζ* is the drag coefficient and 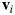 is the velocity at which the actin node at position 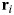 moves in the absence of friction to restore mechanical equilibrium, i.e.
(12)

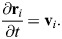
In the simulation, we account for the friction forces (Eqns 11 and 9). Thus, the evolution of the actin node position is given by:
(13)

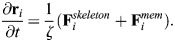
Fig. S1D shows the flowchart of the simulation. This simulation framework was implemented in MATLAB and we used it to investigate different scenarios (see Materials and Methods for details).

In the following sections, we first used a patch of membrane to introduce the different components of the model, namely, spectrin, myosin dynamics and membrane bending. This patch representation facilitates the investigation of how the membrane skeleton responds to mechanical stresses. Then, we investigated how the stochastic dynamics induced by myosin behave on a fully connected network representing the whole cell and proposed mechanisms that enhance cell shape stability.

### Coupling of membrane bending with the actin–spectrin meshwork is important for resisting isotropic contractility

**Fig. 2.**
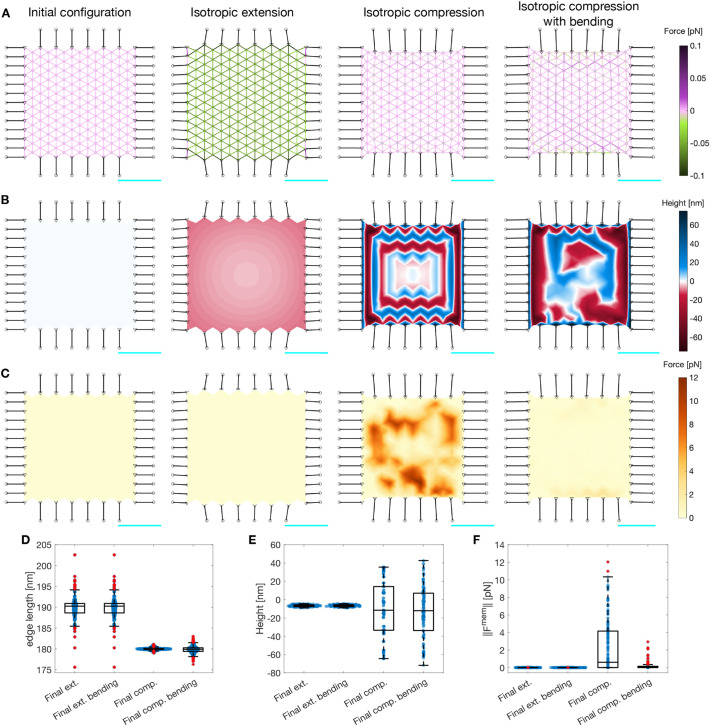
**Actin–spectrin meshwork under symmetrical extension and compression.** (A) Configuration of the meshwork initially and after 180 s of isotropic extension, compression and compression including the force generated by membrane bending. The edges corresponding to spectrin bundles are color coded for the force generated by their spring element. The black edges represent connecting edges and the black circles are focal adhesion nodes with −10 nm height. The black triangles are the nodes linking the connector edges to focal adhesions and spectrin edges. The gray nodes show the locations of short F-actin, which have an initial *z*-axis position of 0 nm. (B) The meshwork as shown in A, color coded for the *z*-axis position of the F-actin nodes. (C) The meshwork as shown in A, color coded for the magnitude of the force generated by the membrane (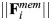). Here 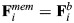. See Materials and Methods for simulation details and Movies 1–3 for the evolution of the meshwork. In A–C, scale bars in cyan correspond to 1 μm. (D–F) Box plots of spectrin edge lengths (D; for each group, *n*=509), F-actin node heights (E; for each group, *n*=150) and magnitudes of the force generated by the membrane (F; for each group, *n*=233) under the different conditions of A–C. Box plots show the median (central line), interquartile (25–75th percentile) range (IQR; boxes), ±1.5×IQR (whiskers) and outliers (red plus symbols).

The membrane skeleton has load-bearing features, which allow cells to resist different deformations. Hence, we tested whether an actin–spectrin meshwork alone can efficiently respond to different imposed stresses. We first simulated the isotropic extension and compression of the actin–spectrin meshwork (module 1, without spectrin unbinding and rebinding) in the absence of any forces generated by the membrane. We introduced a new type of spring edge (module 2) that connects the periphery of the actin–spectrin meshwork to fixed nodes representing focal adhesions in the extracellular space (Fig. S1A, black lines and circles). These connector edges are linked to spectrin edges through protein complexes represented by nodes that update their position according to Eqn 13. Therefore, the nodes linking the connector edges with spectrin edges are differentiated from the short actin nodes (Fig. 2A, black triangles). Note that we chose a fully extended triangular mesh as the initial configuration of the actin–spectrin meshwork. Although this configuration resembles the RBC membrane skeleton configuration, we chose it because it allowed us to have a steady-state initial configuration (i.e. all spectrin edges have the same length) and quantify the deformations induced by mechanical stresses.

We first simulated isotropic expansion. In this case, the connecting edges were pre-extended before the simulation, i.e. we set the initial length *d*_*I*,*C*1_ to be larger than the resting length *d*_0,*C*1_. This way, the connecting edges shrink during the simulation, increasing the contractile force and the length of the spectrin edges (Fig. 2A,D). This results in the expansion of the actin–spectrin meshwork. Note that the *z*-axis position of the F-actin nodes, i.e. their height, decreases at the sides, producing a concave shape of the meshwork (Fig. 2B). To simulate the compression of the meshwork, we set the initial length of the connecting edges to be smaller than the resting length. We observed shrinkage of the meshwork with a drastic change in the *z*-axis position of the F-actin node locations connecting the spectrin edges (Fig. 2B,E). Note that the length of spectrin edges slightly diverges from *d*_0,*S*_ (Fig. 2D). Therefore, we concluded that the meshwork responded to the isotropic compression by changing the *z*-axis position of F-actin nodes instead of the length of spectrin edges. We determined that the response was induced by the initial difference in the *z*-axis position between the actin–spectrin meshwork and the focal adhesion nodes. Moreover, we observed that the magnitude of bending force generated by such height fluctuations (i.e. 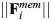, Fig. 2C,F) was high. We concluded that these fluctuations were physiologically unfeasible because they require high amounts of bending energy and the actin–spectrin meshwork by itself was unable to capture isotropic contractility.

Next, we added the membrane bending force (module 3) to balance the force generated by the spectrin and connector springs (see Materials and Methods). The addition of the bending force to the force balance eliminated the large height fluctuations in the actin–spectrin meshwork (Fig. 2B). We observed that the *z*-axis position of more F-actin nodes were closer to the initial height (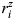
=0 nm) instead of the extreme heights seen in the case without bending (Fig. 2E). Moreover, adjacent nodes had smaller height differences (Fig. S2). As expected, the final configuration minimized the membrane bending energy (Fig. 2C,F). We also found that the bending energy affected the final length distribution of spectrin edges and, therefore, its elastic energy (Fig. 2A,D). However, the addition of the bending energy did not affect the dynamics of the actin–spectrin meshwork under isotropic extension because the difference in *z*-axis position of actin nodes had a slow and smooth evolution (Fig. 2D–F). Thus, our simulations predict that the membrane bending energy interacts with the actin–spectrin meshwork to avoid drastic changes in its configuration when contractile stresses are applied. Furthermore, this interaction minimizes the membrane and spring forces of the actin–spectrin meshwork, resulting in a more efficient physiological response to stresses.

### Unbinding of spectrin edges lowers stresses due to shear deformation

**Fig. 3.**
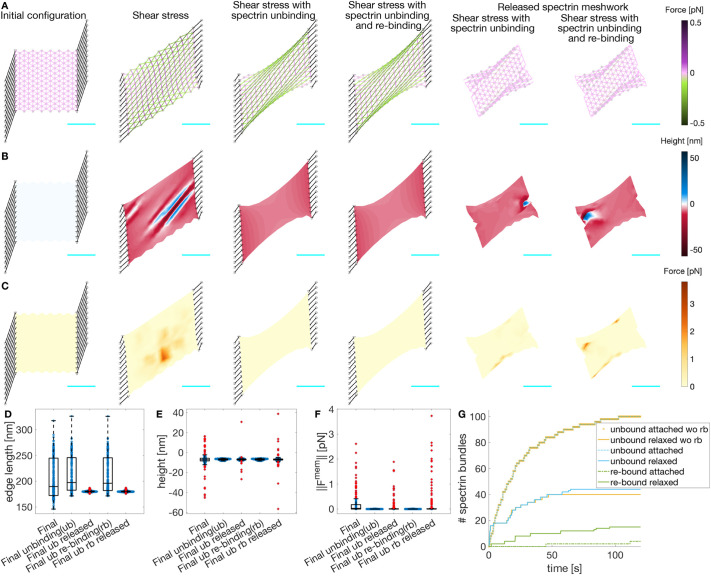
**Actin–spectrin meshwork under shear stress.** (A) Initial configuration and the configuration after 120 s under shear stress, allowing only unbinding of spectrin edges, and allowing unbinding and rebinding of spectrin edges. The last two columns correspond to the case when the meshwork is released from adhesions and evolved for an additional 120 s. Edges are color coded for the force generated by the spectrin spring element. Black lines denote connecting edges and black circles represent fixed focal adhesions with −10 nm height. The F-actin nodes (gray dots) and linker nodes (black triangles) have an initial height of 0 nm. (B) The meshwork as shown in A but color coded for *z*-axis positions of actin nodes. (C) The meshwork as shown in A but color coded for the magnitude of the force generated by the membrane. See Movies 4–6 for the full evolution of the meshwork. In A–C, scale bars in cyan correspond to 1 μm. (D–F) Box plots of the distribution of spectrin edge lengths [D; ‘Final’, *n*=509; ‘Final unbinding (ub)’, *n*=409; ‘Final ub released’, *n*=369; ‘Final ub rebinding (rb)’, *n*=413; ‘Final ub rb released’, *n*=384], F-actin node heights (E; for each group, *n*=162) and magnitudes of the force generated by the membrane (F; for each group, *n*=221) under different conditions. Box plots show the median (central line), IQR (boxes), ±1.5×IQR (whiskers) and outliers (red plus symbols). The median length of the spectrin edges (D) for ‘Final ub released’ is 179.9899 nm and for ‘Final ub rb released’ is 179.8889 nm. A Wilcoxon signed-rank test failed to reject the null hypothesis that the median of the length minus the resting length is equal to zero, with a *P*-value of 0.0773 for the ‘Final ub released’ distribution and 0.9582 for the ‘Final ub rb released’ distribution. Thus, the distribution of the spectrin edge lengths in these cases is significantly equal to the resting length. (G) Cumulative sum of the number of unbound and rebound spectrin edges over time with or without rebinding (‘wo rb’).

In cells, spectrin supports in-plane shear deformation (Leterrier and Pullarkat, 2022). Hence, we investigated whether our actin–spectrin meshwork with membrane forces can withstand such deformation. To mimic the stress, we removed the adhesions parallel to the *x*-axis and changed the position of the adhesions parallel to the *y*-axis as follows. On the right end of the meshwork, the adhesions were located 360 nm from the linking nodes (black triangles in Fig. 3A) in the *x*-direction and 1080 nm in the *y*-direction. On the left end of the meshwork, the adhesions were located at similar distances in the *x*- and *y*-direction, but with opposite polarity. As the initial length of the connecting edges (*d*_*I*,*C*3_) was larger than their resting length (*d*_0,*C*3_), the connecting edges were pre-extended. This configuration guaranteed that during the simulation, the meshwork would extend in one direction and be compressed in the orthogonal direction (Fig. 3A–C). At the end of the simulation, we observed high fluctuations in the *z*-axis position of short F-actin nodes at the center of the meshwork (Fig. 3B), which resulted in high membrane bending force (Fig. 3C). Moreover, the meshwork was under high contractile and expansive forces (Fig. 3A). However, experimental evidence shows that the spectrin network in RBCs can deform and experience high shear stress (An et al., 2002). To do so, spectrin tetramers dissociate to dimers when a low shearing force is applied (An et al., 2002). Therefore, we included this mechanism by removing the spectrin edges that generated an expanding force greater than a threshold force (module 2), as in Ghisleni et al. (2024).

Fig. 3B,C shows that the F-actin height fluctuations and the magnitude of the membrane force were reduced when spectrin unbinding was included in the meshwork dynamics. Furthermore, the final configuration of the meshwork was shrunk along the long axis with large spectrin edges (Fig. 3A). Note that most spectrin removal occurred within the first 30 s of the simulation (Fig. 3G, dotted yellow line). Towards the end of the simulation, the spectrin edge energy was reduced, reaching a quasi-steady state with little to no spectrin edge removal. Spectrin unbinding reduces mechanical stress, but can the meshwork recover its shape after eliminating the stresses? To test this, we detached the connecting edges from the actin–spectrin meshwork in Fig. 3A and simulated the model for an additional 120 s. Note that the meshwork reached a new steady state after 40 s with reduced spectrin edge removal (Fig. 3G, yellow solid line). In this new steady state, the resting length of spectrin edges was recovered, thereby minimizing the meshwork stress (Fig. 3A,D). Note that the *z*-axis positions of actin nodes showed some fluctuations at the end of the simulation, which might result from the force balance (Fig. 3B,E). Such fluctuations also affected the force generated by the membrane (Fig. 3B,F). Overall, the actin–spectrin meshwork remained clustered on the long axis sides and did not recover its shape.

We next investigated whether spectrin rebinding would change the response of the meshwork. We allowed the unbound spectrin edges to rebind when the distance between the two F-actin nodes to which an edge was connected was equal to or larger than the resting length. Fig. 3A–C shows that the evolution of the meshwork with spectrin unbinding and rebinding is similar to that of the meshwork with only unbinding. Moreover, the evolution of the total number of spectrin unbound edges was similar when the meshworks were attached to the focal adhesions (Fig. 3G, dotted yellow and blue lines). The resulting meshworks only differed when released from the focal adhesions: the meshwork allowing spectrin edge rebinding showed more unbinding events (Fig. 3G, solid yellow and blue lines). As expected, spectrin rebinding events were fewer when the meshwork was attached to focal adhesions, but the events increased when it was released (Fig. 3G, green). However, when the meshwork reached a steady state, i.e. the length of spectrin edges was equal to the resting length, the unbinding and rebinding events ceased, and the meshwork remained clustered. Hence, we hypothesized that additional mechanisms are needed to prevent spectrin clustering after the stress is removed, thereby promoting spectrin redistribution in the cell to provide mechanical support to the membrane and bear future stresses at different locations.

### Myosin interactions with the actin–spectrin meshwork promote recovery of spectrin edges

We next asked under what conditions would the membrane skeleton recover a prestressed configuration after the external loading is removed. Recent work has shown that spectrin topological transitions are driven by actomyosin contractility (Ghisleni et al., 2024). Therefore, we incorporated the dynamics of myosin into the meshwork (module 4). As in Ghisleni et al. (2024), myosin edges generated a contractile force and were removed when they shrunk to a minimal length or when there were no available binding sites. In addition to these dynamics, myosin edges were added and removed stochastically (Baird et al., 2017).

**Fig. 4.**
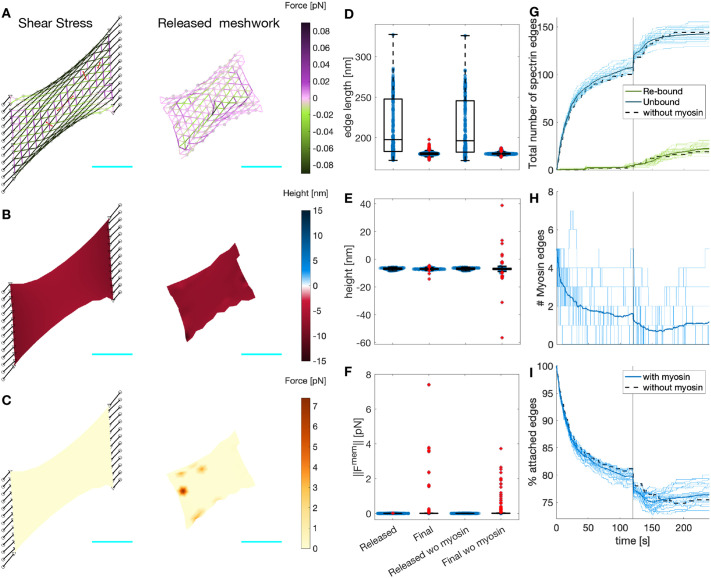
**Myosin dynamics on an actin–spectrin meshwork under shear stress.** (A) Meshwork configuration after 120 s under shear stress and at 120 s after releasing the network from the focal adhesion nodes. Edges are color coded for the force generated by the spectrin spring element. Black lines denote the connecting edges and black circles denote fixed focal adhesions with −10 nm height. Red edges correspond to myosin. Gray circles represent F-actin nodes and black triangles represent linker nodes with an initial *z*-axis position of 0 nm. (B) The meshwork as shown in A but color coded for actin node height. (C) The meshwork as shown in A but color coded for the magnitude of the force generated by the membrane. See Movie 7 for the full evolution of the meshwork. In A–C, scale bars in cyan correspond to 1 μm. (D–F) Box plots of spectrin edge lengths [D; ‘Released’, *n*=407, ‘Final’ *n*=390, ‘Released without (wo) myosin’, *n*=413; and ‘Final wo myosin’, *n*=384], actin node heights (E; for each group, *n*=162), and membrane force magnitudes (F; ‘Released’, *n*=237; ‘Final’ *n*=237; ‘Released wo myosin’, *n*=221; and ‘Final wo myosin’, *n*=221) for the configurations shown in A–C. The values for the meshwork without myosin (Fig. 3) are given for comparison. Box plots show the median (central line), IQR (boxes), ±1.5×IQR (whiskers) and outliers (red plus symbols). (G) Evolution of the total number of unbound (blue) and rebound (green) spectrin edges. (H) Evolution of the number of myosin edges. (I) Evolution of the percentage of attached spectrin edges. In G–I, the thin lines correspond to 31 different simulations, the thick line is the temporal average of the simulations, and the black dashed line shows the evolution of the meshwork without myosin as in Fig. 3. In G, the mean value of the total number of rebound spectrin edges at the end of the simulation (22.4333) was significantly different (one-sample two-tailed *t*-test, *P*=5.2923×10^−5^) than the end value of the simulation without myosin (19), whereas the total number of unbound spectrin edges at the end of the simulation (142.5667) was not significantly different (one-sample two-tailed *t*-test, *P*=0.2687) than the end value of the simulation without myosin (144). In I, the mean value of the percentage number of attached spectrin edges at the end of the simulation (76.3982) was significantly different (one-sample two-tailed *t*-test, *P*=0.0026) than the end value of the simulation without myosin (75.4420).

We observed that the lengths of spectrin edges and *z*-axis positions of actin nodes were similar to those of the meshwork without myosin (Fig. 4A–F). However, myosin increased the rebinding events after the meshwork was released (Fig. 4G). Due to the stochastic nature of the myosin dynamics, we ran 30 additional simulations to test the generality of the results and obtained statistics. We observed that after the initial 60 s, the number of myosin edges in the system with a surface area of ∼2.5 μm^2^ settled to one (Fig. 4H), which promoted unbinding and rebinding in the spectrin meshwork (Fig. 4G). Thus, a single myosin edge per 2.5 μm^2^ was enough to promote spectrin edge turnover. In some simulations, the final percentage of attached spectrin edges matched the percentage of attached edges before releasing the spectrin meshwork from focal adhesions (Fig. 4I). We concluded that myosin addition avoids the clustered, crumpled state and helps meshwork recovery, which prepares the membrane skeleton to respond to new stresses.

**Fig. 5.**
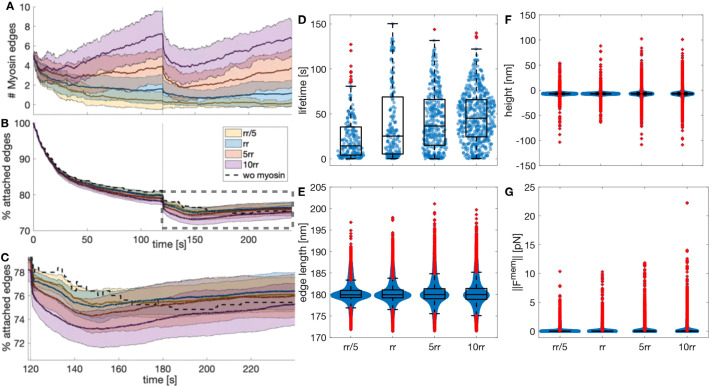
**Myosin dynamics under different stochastic addition and removal rates.** (A) Temporal evolution of the myosin edges in the actin–spectrin meshwork with different ratios of addition and removal rates (*rr*=*ϕ*_*a*_/*ϕ*_*b*_), color coded as in B. The values correspond to *rr*/5, *rr*, *5rr* and 10*rr*. The thick line represents the mean and the shadowed area is the standard deviation from 31 simulations. The black vertical line denotes the time of network release from shear stress. (B) Temporal evolution of the percentage of attached spectrin edges over time. (C) Magnified image of the dashed rectangle in B. In B,C, the black dashed line shows the evolution of the meshwork without myosin, as in Fig. 3. (D–G) Box plots of myosin edge lifetimes (D; for *rr*/5, *n*=212; for *rr*, *n*=193; for 5*rr*, *n*=362; and for 10*rr*, *n*=553), spectrin edge lengths (E; for *rr*/5, *n*=11,622; for *rr*, *n*=11,666; for 5*rr*, *n*=11,604; and for 10*rr*, *n*=11,495), F-actin node heights (F; for each group, *n*=4860) and magnitudes of the force generated by the membrane (G; for *rr*/5, *n*=7064; for *rr*, *n*=7086; for 5*rr*, *n*=7578; and for 10*rr*, *n*=8142) at the end of the simulation for different *rr* values. Box plots show the median (central line), IQR (boxes), ±1.5×IQR (whiskers) and outliers (red plus symbols).

Next, we investigated whether increasing the number of myosin edges acting in the actin–spectrin meshwork enhanced the rebinding of spectrin edges and, thereby, meshwork recovery after removing the stress. For this, we changed the ratio of rates corresponding to the random addition and removal of the myosin edges. This rate ratio (*rr*) is given by:
(14)

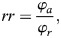
where *ϕ*_*a*_ and *ϕ*_*r*_ are the rates for random addition and removal of myosin edges, respectively. We found that increasing and decreasing the rate ratio resulted in more and less myosin edges in the simulations, respectively, even after releasing the meshwork from the focal adhesions (Fig. 5A). Moreover, increasing *rr* raised the median and reduced the spread of the lifetimes of the myosin edges (Fig. 5D). Thus, the higher the stochastic addition-to-removal ratio, the more myosin edges exert contraction at different zones of the actin–spectrin meshwork. Paradoxically, this hinders the contraction of the myosin edges and their removal when they reach a minimal length, extending the edge lifetime but preventing the creation of space for spectrin edges to rebind. On average, both increasing and reducing *rr* resulted in a smaller increase in the percentage of attached spectrin edges (Fig. 5B,C), suggesting that there is an optimum number of myosin edges acting on the meshwork that allows further rebinding of spectrin edges.

We examined the final configuration of the simulations. We found that the spectrin edge lengths (Fig. 5E) and *z*-axis positions of actin nodes (Fig. 5F) were less spread for the original parameters than for the increased *rr*. For the actin node height, the interquartile range (IQR) was 0.9242 nm for *rr*, 1.3378 nm for 5*rr* and 1.3324 nm for 10*rr*. For the spectrin edge length, the IQRs were 1.8201 nm (*rr*), 2.3228 nm (5*rr*) and 2.5261 nm (10*rr*). With larger *rr*, the spread of the magnitude of the membrane force also increased from an IQR of 0.0158 pN to 0.0398 pN for 5*rr* and 0.0339 pN for 10*rr* (Fig. 5G). More myosin edges in the meshwork did not improve the recovery of spectrin edges and produced stress in the meshwork, i.e. the spectrin edge length deviated more from the resting length and the height of the actin node was more divergent, which exerts spring and bending energy. When decreasing the parameters to *rr*/5, the IQRs for spectrin length (1.6163 nm), actin node height (0.9195 nm) and membrane force magnitude (0.0150 pN) were similar to the original values. However, the difference between the mean percentage of attached edges at the time when the meshwork is released and at the end time was higher for *rr*/5 (3.8048%) than for *rr* (3.2809%). Thus, the meshwork with a smaller rate ratio had a slightly worse recovery. We concluded that our original parameters, which resulted in a single myosin per 2.5 μm^2^ acting on the meshwork after releasing it from focal adhesions, gave a more efficient recovery. These qualitative findings suggest that cells use the required number of myosins to enhance cytoskeletal recovery after inducing stress and that this function is tightly regulated.

### Cell adhesion promotes actin–spectrin meshwork stabilization and conserves cell shape

**Fig. 6.**
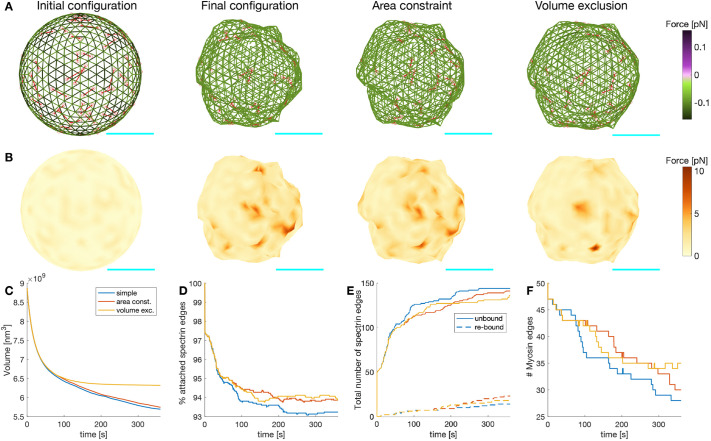
**Actin–spectrin meshwork dynamics in a suspended cell.** (A) Initial configuration of the actin–spectrin spherical meshwork and 360 s of the simulation, with area constraint of the membrane and volume exclusion of the cytoplasm. Edges are color coded for the force generated by the spring potential energy of the spectrin edges. Red lines correspond to myosin edges. (B) The meshwork as shown in A but color coded for the force generated by the membrane. Here, 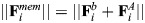. See Movies 8–10 for the full evolution of the meshwork. In A,B, scale bars in cyan correspond to 1 μm. (C–F) Graphs showing time evolution of the volume (C), percentage of attached spectrin edges (D), total number of unbound and rebound spectrin edges (E) and number of myosin edges (F).

Cells in suspension and adhered cells have different mechanical properties (Dabbiru et al., 2023). Therefore, we next investigated how the actin–spectrin meshwork differs in geometries similar to those of cells in suspension, such as RBCs, and of cells with adhesions, such as fibroblasts. To do this, we implemented the model on a fully connected meshwork. For cells in suspension, we chose a sphere to capture the simplest fully connected 3D shape and avoid computational challenges associated with high curvatures. In this case, unlike the meshwork resembling a patch of membrane, the initial configuration of the spectrin edges was under stress due to deviations of their lengths from the resting length (Fig. 6A). Such deviations were necessary to obtain a spherical shape. The spectrin edges with an initial length smaller than the resting length generated a high shrinking force, in some cases, above the threshold force (*F*^*th*^). Therefore, they were removed shortly after the start of the simulation (blue lines, Fig. 6D,E). After 360 s of the simulation, we observed that the sphere crumbled (Fig. 6A) and increased its membrane force (Fig. 6B), while dynamically adding and removing spectrin edges (blue lines, Fig. 6D,E). Moreover, the sphere volume (blue line, Fig. 6C) and the number of myosin edges (blue line, Fig. 6F) showed a sustained decrease, arising from the myosin contractile action. However, experimental data show that myosin contractility maintains cell shape (Smith et al., 2018). Thus, we hypothesized that there must be further mechanisms that guarantee cell shape maintenance. Such mechanisms should be linked to the cell shape and signal when cells go through a sustained shrinkage or expansion in the absence of induced mechanical forces.

As in Alimohamadi et al. (2020), we assumed that the plasma membrane resists stretching. Indeed, experiments show that high stretching moduli are conserved for different types of lipid bilayers (Rawicz et al., 2000) and, hence, local membrane incompressibility can be assumed (Alimohamadi et al., 2020). This was implemented in the model by adding a surface area constraint to the force generated by the membrane, now 
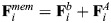
 (see module 3). We found that when the surface area of the plasma membrane was constrained, the number of unbound spectrin edges was reduced and rebinding of these edges was promoted (Fig. 6E). Thus, the percentage of attached spectrin edges was higher (Fig. 6D). We also observed that the number of myosin edges was higher (Fig. 6F), which resulted from the change in the force balance that hindered the myosin contraction and, thereby, their removal. The locations where the force generated by the membrane was high before implementing surface area constraint to the force balance were smoothed out, thereby reducing the crumbled appearance.

The size of the sphere under surface area constraint of the plasma membrane was bigger but its volume kept decreasing (Fig. 6C). It is known that non-dividing adult cells maintain their size (Lloyd, 2013) and, based on experimental data, we only expect volume fluctuations in the absence of any stimulus at the simulation timescale (Roffay et al., 2021). Hence, we implemented volume exclusion in the model to represent the presence of organelles in the cytosol by restricting the movement of actin nodes to a volume 15% smaller than the initial volume (module 3). Based on experiments where hyperosmotic shocks caused a non-reversible volume decrease (Roffay et al., 2021), we assumed that larger volume deviations trigger further cellular and biochemical processes. A simulation with surface area constraint of the plasma membrane and volume exclusion of the cytoplasm showed that the sphere settled to a steady volume (Fig. 6C) while experiencing spectrin edge unbinding and rebinding events (Fig. 6E). Moreover, the force generated by the membrane was reduced (Fig. 6B). Therefore, we concluded that the interaction between the actin–spectrin meshwork and the membrane constrained by surface area and volume exclusion promoted shape integrity. Although the actin–spectrin meshwork allows cells in suspension to deform and bear different stresses, the membrane surface area constraint and volume exclusion of the cytoplasm guarantee shape integrity. As the evolution of the actin node positions, dictating the cell shape, is governed by the force balance between the membrane skeleton forces (Eqn 9), and adding surface area constraint and volume exclusion enhances the shape integrity, we conclude that the interaction between these elements benefits the overall dynamics. Thus, our model hints at a feedback mechanism between the membrane and the skeleton that goes beyond the accepted function of the spectrin skeleton in passively giving mechanical support to the membrane (Li et al., 2023). Such a mechanism needs to account for the global state of the cell to maintain the steady-state shape.

**Fig. 7.**
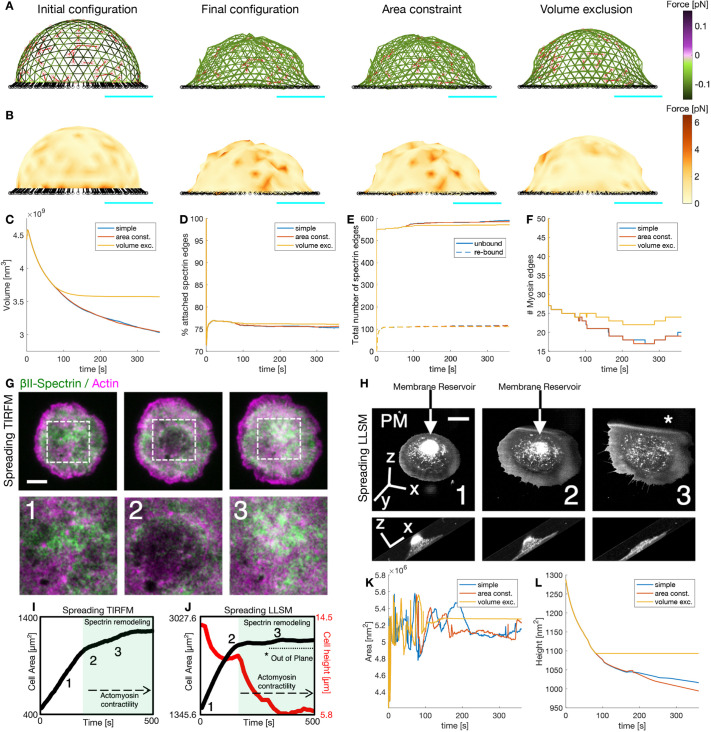
**Actin–spectrin meshwork dynamics in an adhered cell.** (A) Initial configuration of the actin–spectrin meshwork in an adhered cell and after 360 s of the simulation, with area constraint of the plasma membrane and volume exclusion of the cytoplasm. Edges are color coded for the force generated by the spring potential energy of the spectrin edges. Red lines correspond to myosin edges. The black lines represent connecting edges and black circles represent connecting nodes. (B) The meshwork as shown in A but color coded for the force generated by the membrane. Here, 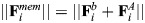. See Movies 11–13 for the full evolution of the meshwork. In A,B, scale bars in cyan correspond to 1 μm. (C–F) Time evolution of the volume (C), percentage of attached spectrin edges (D), total number of unbound and rebound spectrin edges (E) and number of myosin edges (F). (G) Cell spreading analysis at the cell body (zooms corresponding to the dashed white boxes), displayed by live total internal reflection fluorescence microscopy (TIRFM) images (green, GFP–βII-spectrin; magenta, RFP–actin). Relevant events observed between independent experiments are shown (1–3), in particular, endogenous actin node formation and corresponding βII-spectrin behavior. Scale bar: 10 μm. Figure adapted from figure 4a in Ghisleni et al. (2020) under the terms of a CC BY 4.0 license. (H) Cell spreading imaged by lattice light-sheet microscopy (LLSM) in mouse embryonic fibroblasts transfected with the membrane reporter Scarlet-PM(Lck). The relevant frames 1–3 are reported in the orthogonal views (whole cell) and in the lateral projections (to highlight cell height). The membrane reservoir is on the top of the cell body and dissolved during the slow-growth phase of spreading (P2). See Movie 14. Scale bar: 10 μm. (I) Projected cell area analysis over time and the relative positioning of frames 1–3 presented in G are shown in the graph. Activation of actomyosin contractility and spectrin remodeling during the slow-growth phase of spreading (P2) is highlighted in green. This is a new analysis of data obtained as part of a previously published study (Ghisleni et al., 2020). (J) Projected cell area (black) and cell height (red) analysis over time and the relative positioning of frames 1–3 presented in H are shown in the graph. Activation of actomyosin contractility and spectrin remodeling during the slow-growth phase of spreading (P2) is highlighted in green and correlates to the flattening of the cell body. The portion of the cell that is excluded from the illumination plane is indicated by the asterisk in H. (K,L) Time evolution of the projected cell area (K) and cell height (L) for the simulation.

Most cells are embedded in the extracellular matrix and adhere to it, which alters the actin–spectrin meshwork. Therefore, we examined the meshwork dynamics in a configuration that resembles a cell adhered to a surface. We took the initial sphere configuration of Fig. 7A and set *r*^*z*^=0 for all the F-actin nodes in the south hemisphere, i.e. locations with *r*^*z*^<0. Then, we added springs as connecting edges to attach the F-actin nodes at position (*r*^*x*^, *r*^*y*^, *r*^*z*^=0) to fixed nodes located at (1.1*r*^*x*^, 1.1*r*^*y*^, −100 nm). Such an arrangement guaranteed that the initial length of the linker springs (*d*_*I*,*C*4_) was larger than the resting length (*d*_0,*C*4_). Thus, the bottom of the hemisphere was stretched during the simulation, inducing a shape change (Fig. 7A,B). We simulated the same cases as in the sphere (Fig. 6A,B) and observed that the final configuration was less crumpled when considering area constraint and volume restriction. Moreover, the spectrin edge and membrane forces were reduced and the volume stabilized (Fig. 7A–C). The spectrin edges experienced a rapid detachment after the start of the simulation, induced by the contraction of the connecting edges (Fig. 7E). However, the percentage of attached spectrin edges immediately stabilizes (Fig. 7D). Interestingly, the number of myosins in the meshwork of the adhered cell settled to a mean value earlier than in the suspended configuration (Fig. 7F). Altogether, we found that the actin–spectrin meshwork was more stable when connected to the substrate. We hypothesize that when cells adhere, the actin–spectrin meshwork stabilizes to organize membrane proteins (Teliska and Rasband, 2021).

Cell spreading represents an active biological process in which adhesion to the substrate, membrane remodeling and cytoskeletal modifications simultaneously occur and interplay (Gauthier et al., 2011). More specifically, previously published total internal reflection microscopy data (Ghisleni et al., 2020) and novel observations by high temporal-resolution lattice light-sheet microscopy (LLSM) highlighted how spectrin remodeling is driven by the reawakening of actomyosin contractility (Fig. 7G–J). Interestingly, this slow-growth phase of spreading (also referred to as P2) corresponded to the exhaustion of the membrane reservoir (area constraint) and the flattening of the cell body towards an equilibrium state (volume constraint) highlighted by the four-dimensional LLSM imaging approach (Fig. 7H,J). Our modeled adhered cell that considers membrane area constraint and volume exclusion showed a hindered increase in projected cell area (Fig. 7K) and it flattens over time (Fig. 7L). These correlative observations closely resemble the series of events captured by our model, suggesting that the enhanced stability of the adherent meshwork is important for cell function.

## DISCUSSION

Using a model of the spectrin skeleton, we examined possible mechanisms for cells to bear different stresses, with a focus on investigating the stability of cell shape. Although previous models of spectrin with molecular details have been proposed (Jäger et al., 2022; Li et al., 2005; Peng et al., 2013; Gov and Safran, 2005; Li and Lykotrafitis, 2014), these models do not offer a comprehensive investigation of the mechanisms for cell shape stability and remodeling. Our simulations revealed the following outcomes, relevant to the biophysics of the actin–spectrin meshwork. First, the plasma membrane is critical in lowering fluctuations in the actin–spectrin meshwork, hinting at an active role for the interaction between the plasma membrane and actin–spectrin meshwork rather than the experimentally studied passive function of spectrin skeleton in maintaining the stability and structure of the plasma membrane (Teliska and Rasband, 2021). This is in line with interactions observed in other models containing a molecular description of spectrin (Li et al., 2005, 2007; Peng et al., 2013; Li and Lykotrafitis, 2014). We tested possible mechanisms that promote the actin–spectrin meshwork response to different stresses and the meshwork recovery after the stresses are removed, such as spectrin unbinding and rebinding and stochastic dynamics of myosin. These mechanisms are difficult to examine in experiments *in vivo* owing to technical restrictions. Having characterized the responses of the membrane skeleton to induced stresses in a patch, we investigated how suspended cells can remain at a stable size despite constant fluctuations induced by myosin dynamics, which are necessary for responding to imposed stresses. We proposed that cells must have a readout of their surface area and volume and induce forces when the cell deviates from its steady-state shape. Finally, we modeled adhered cells, which have different mechanical properties than suspended cells (Dabbiru et al., 2023). We related our *in silico* findings with our published (Ghisleni et al., 2020) and unpublished data (A.G., unpublished), which show that the cell size is maintained after depletion of membrane reservoir and there is flattening of the cell body despite the spectrin remodeling driven by myosin contractility. Our model focuses on the mechanical properties of the spectrin skeleton, which we assumed to be shared between different types of cells, and is based on experimental observations in RBCs and fibroblasts. Thus, our comparisons with experimental data are only qualitative. A quantitative comparison would require taking the parameters from experimental data for a specific cell type and perhaps adding cell-specific elements to the model.

Specifically, we showed that bending energy from the plasma membrane and spectrin detachment is necessary to bear isotropic compression and shear stress. We assumed that the bending energy from the plasma membrane is transmitted to the actin–spectrin meshwork via the short actin nodes, as in Li et al. (2005). Although more sophisticated descriptions for the link between the skeleton and lipid membrane have been proposed (Peng et al., 2013; Li et al., 2018), our chosen description of the bending energy reduces the fluctuations in the *z*-plane of a simulated meshwork patch. Experimental evidence for dissociation of spectrin tetramers into dimers under shear response, which can relate to spectrin edge unbinding, has been available for a long time (An et al., 2002). However, only recently have the changes in the number of bound spectrin to short F-actin complexes been examined using a theoretical model (Ghisleni et al., 2024; Fai et al., 2017; Chai et al., 2023). In RBCs, experimental and theoretical efforts have described how the ATP-driven flickering of the membrane can be related to spectrin skeleton remodeling (Rodríguez-García et al., 2015; Park et al., 2010; Turlier et al., 2016), thereby connecting RBC deformation to spectrin morphology.

In this work, we improved the 2D model in Ghisleni et al. (2024) by considering the rebinding of the unbound spectrin edges to test whether the system can return to the initial state after the stress is removed. Due to a lack of experimental evidence for the rebinding mechanism of spectrin bundles, we chose the simplest rule: edges rebind to the same actin nodes when the distance between the nodes is equal to the resting distance. Other rules have been proposed; for example, a model of RBCs with the stochastic addition and removal of spectrin edges shows that repeated deformations will lead to structural changes in the cytoskeleton (Fai et al., 2017). Future theoretical efforts can explore different rules for spectrin rebinding and the effects on the connectivity of the actin–spectrin meshwork. Furthermore, buckling of spectrin edges can be considered as in a recent model of a network of fibrin fibers, which shows the importance of buckling for describing shear response in the network (Zakharov et al., 2024).

In our simulations, the skeleton with membrane bending energy and unbinding and rebinding of spectrin edges settles to a clustered steady state when the spectrin edges recover their resting lengths. However, we hypothesized that, after removing the stress, the spectrin meshwork connectivity should recover. Based on the interaction between myosin and the actin–spectrin meshwork observed in fibroblasts (Ghisleni et al., 2024) and RBCs (Smith et al., 2018), we added myosin to the network. As in Ghisleni et al. (2024), we assumed that myosin edges are contractile until they reach a minimum length and are removed from the network. Moreover, myosin edges are stochastically added and removed, mimicking the spatially heterogeneous contribution of myosin to cell contractility. These assumptions resulted in a more dynamic actin–spectrin meshwork, which showed an enhanced recovery from the stress. Although theoretical models have studied aster formation and stabilization of stress fibers due to myosin action (Vernerey and Akalp, 2016), this consideration is out of scope for our model because short actin filaments are represented as nodes. Thus, our model only considers a pulsative action of myosin, as reported in Ghisleni et al. (2024). In our simulations, we found an optimum balance between the stochastic addition and removal rates of myosin edges. Experiments could test whether increasing or decreasing the number of myosin rods acting on the actin–spectrin meshwork enhances its response after shear stress is induced. It has been shown that RBCs contain ∼150 non-muscular myosin IIA bipolar filaments per cell (Alimohamadi et al., 2020; Smith et al., 2018). Although a previous model of the RBC cytoskeleton considers myosin forces (Alimohamadi et al., 2020), it uses a deterministic description to inform the stable configurations. In our model, myosin gives a stochastic feature that allows a continuous reconfiguration of the actin–spectrin meshwork, as observed in live-cell imaging experiments (Ghisleni et al., 2020, 2024).

Next, we showed that despite the continuous stochastic dynamics of the skeleton, a fully connected meshwork can reach a stable state with a given volume and fluctuating number of spectrin and myosin edges. To keep the generality of our approach, we tested two cases that resemble cells with different properties: suspended and adherent cells. For this, we considered surface area conservation of the plasma membrane and volume exclusion of the cytoplasm due to the presence of different molecules and organelles. Moreover, the actin–spectrin meshwork stabilizes sooner in the adhered case. Future efforts can consider how the distribution of spectrin and myosin edges changes after different stresses are applied in different cells.

The parameters related to spectrin and myosin mechanical properties were taken from Ghisleni et al. (2024). These model parameters were fitted to replicate experimental observations instead of taking values from the literature, which differ depending on cell type and experimental settings. Future efforts could change these fitted mechanical parameters to the experimental values for different types of cells and, thereby, investigate whether the characteristic scales change.

In our model, we assumed that the meshwork is dynamic even when it is not stressed, in line with experimental evidence (Nowak et al., 2022). To simulate such a dynamic meshwork, we chose a simplified representation of spectrin bundles as Hookean springs. Spectrin bundles are usually represented using a worm-like chain (WLC) model (Jäger et al., 2022; Boey et al., 1998; Li et al., 2018) to account for thermal fluctuations of polymers (Doi et al., 1988) or interpolation of the WLC (Discher et al., 1998; Li et al., 2005; Dao et al., 2006; Feng et al., 2020; Peng et al., 2013; Alimohamadi and Rangamani, 2023), proposed by Marko and Siggia (1995) and Bustamante et al. (1994), which avoids the collapse of the spectrin bundle under compression and bounds it under expansion, while behaving like an ideal spring at the minimum (Discher et al., 1998). Bundles in WLC models can bend and, thus, networks using a WLC description for fiber edges have given insight into the effect of single-fiber bending rigidity on the network (Broedersz et al., 2011). However, WLC representations of spectrin bundles are highly non-linear and require significant computational power. Alternatively, the simple Hookean spring potential has been used and proved (Svetina et al., 2016) to coincide with the WLC potential used in Discher et al. (1998) and Dao et al. (2006) for small extensions. In our simulations, we controlled the applied stress, which resulted in the extension and contraction of the spectrin edges within the small extension criteria (2*d*_0,*S*_ and 0.6*d*_0,*S*_, respectively) (Svetina et al., 2016). Moreover, the resulting spectrin edges are below the spectrin length when all the repeats are unfolded (≈1022 nm) (Rief et al., 1999). Thus, a Hookean spring representation of the spectrin bundles is well suited for our investigation. This mesoscopic depiction of the membrane skeleton, which omits its molecular details given in other models (Zhang et al., 2017; Jäger et al., 2022; Chai et al., 2023), allows us to examine the overall configuration changes due to the induced stresses. Importantly, we chose this mesoscopic model because we are interested in the effects of dynamically adding and removing, either randomly or due to applied forces, the skeleton components embedded in a membrane. The predictions derived from our model can be tested experimentally; for example, the prediction of our model regarding the optimum number of myosins acting on the actin–spectrin meshwork to promote its recovery after removing externally imposed stresses. Another prediction that could be tested experimentally is that adherent cells are more stable than suspended cells. Future efforts can add more molecular detail to our model.

## MATERIALS AND METHODS

### Simulations

For the simulations in Fig. 2, we solved Eqn 13 with 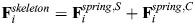 and 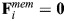 for the isotropic extension and compression, and 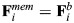 (Eqn 4) when adding bending. The unbinding and rebinding of spectrin edges were implemented first in Fig. 3. Myosin dynamics were introduced in Fig. 4. Hence, in Eqn 13, 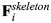 was then defined as in Eqn 10. For the plots showing the *z*-axis positions of F-actin nodes and membrane force, we obtain the values for each F-actin node and use interpolated coloring for the triangular surfaces.

To calculate the spreading of the data contained in the box plot of Fig. 5, we used the IQR, which is defined as the difference between the 75th and 25th percentiles of the data (i.e. the top and the bottom edges of the box). The IQR does not account for the data outliers and gives a better representation of the data range.

For the fully connected sphere meshwork and the adhered cell meshwork in Figs 6 and 7, we took 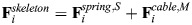 in Eqn 13. Note that when including the area constraint and volume restriction, we defined 
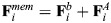
.

### Parameter fit

The parameters used for the spectrin and myosin edges were taken from Ghisleni et al. (2024). As in Vignaud et al. (2021), instead of taking the mechanical parameters (*k*_*s*,*S*_, *k*_*c*,*M*_) from the literature, which vary depending on the cell type and experimental assay, we fitted these parameters to replicate the experimental findings. The spatial and temporal scales were informed by experiments. We set the resting length *d*_0,*S*_ to 180 nm, which coincides with the periodicity of clustered spectrin in Ghisleni et al. (2024). The myosin maximum and minimum lengths (*d*_*max*_ and *d*_*min*_) were based on data in Billington et al. (2013). The time step Δ_*t*_ and drag coefficient *ζ* were fitted so that the actin–spectrin meshwork had a smooth evolution over time. The stochastic addition and removal rates (*ϕ*_*a*_ and *ϕ*_*r*_) were set to match experimental values reported in Ghisleni et al. (2024).

The size of the initial configuration of the actin–spectrin meshwork in Figs 2–5 was chosen based on experimental data in Ghisleni et al. (2024), where patches of spectrin clusters of ∼2.5 μm length were observed. We set the parameters related to the connecting edges (*k*_*s*,*C*_, *d*_0,*C*1_, *d*_0,*C*2_, *d*_0,*C*3_, *d*_*I*,*C*1_, *d*_*I*,*C*2_ and *d*_*I*,*C*3_) to induce structural changes in the actin–spectrin meshwork. The membrane bending and surface area constants (*k*_*b*_ and *k*_*A*_) were taken from Li et al. (2005). When we introduced the area constraint in the suspended cell, we took the sphere as the permanent reference state (i.e. *A*_0_ was the initial area of the sphere), as in Li et al. (2005). Thus, we used their proposed value of *k*_*b*_=200*k*_*B*_*T*, where *k_B_* is the Boltzmann constant and *T* is the temperature. This value is larger than the commonly used values of *k*_*b*_ but results in more stable shapes. To ease the computational burden of the simulations for the suspended and adhered cells, we set the cell radius to 1.25 μm. Note that the cells are approximately on the same scale as that of the actin–spectrin meshworks.

### Numerical implementation

We ran the simulations in MATLAB R2021a on a desktop computer. Following Ghisleni et al. (2024), for the patch of the actin–spectrin meshwork (Figs 2–5), we obtained the initial spectrin mesh with the ‘delaunayTriangulation.m’ function in MATLAB and implement the forward Euler method to solve Eqn 13. We traced the sphere in Fig. 6 with the ‘icosphere.m’ function (https://www.mathworks.com/matlabcentral/fileexchange/50105-icosphere) and used the ‘remeshing.m’ function (https://github.com/christopherhelf/isotropicremeshing) to obtain a (semi)isotropic shape consisting of equilateral triangles with side length *d*_0,*S*_.

### Code availability

The code is available in GitHub (https://github.com/RangamaniLabUCSD/Spectrin-topological-transition) and published in Zenodo (https://zenodo.org/records/14263179).

### LLSM

The lattice light-sheet microscope (Chen et al., 2014) used was developed by Eric Betzig [Howard Hughes Medical Institute (HHMI) Janelia Research Campus] and operated and maintained in the Advanced Imaging Center at the HHMI Janelia Research Campus (Ashburn, VA, USA). 488, 560 or 642 nm diode lasers (MPB Communications) were operated between 40 and 60 mW initial power, with 20–50% acousto-optic tunable filter transmittance. The microscope was equipped with a Special Optics 0.65 NA/3.75 mm water dipping lens, excitation objective, and a Nikon CFI Apo LWD 25×1.1 NA water dipping collection objective, which used a 500 mm focal length tube lens. Live cells were imaged in a 37°C heated, water-coupled bath in FluoroBrite medium (Thermo Fisher Scientific) with 0–5% fetal bovine serum (Euroclone ECS0182L) and penicillin/streptomycin. Mouse embryonic fibroblasts (MEFs) were transfected 24 h before the experiment with the mScarlet-PM (Lck) plasmid (Addgene, 98821). Before the experiment, cells were trypsinized, centrifuged for 5 min at 30 ***g***, washed once with PBS, and serum-starved in suspension for 30 min at 37°C in CO_2_-independent 1× Ringer's solution [1× (≈300 mOsm): 150 mM NaCl, 1 mM MgCl_2_, 1 mM CaCl_2_, 20 mM Hepes (pH 7.4), 5 mM KCl and 2 g/l glucose]. Suspended cells were thereafter kept at room temperature for up to 3 h. Transfected MEFs were added directly to the coverslip submerged in the medium bath prior to acquisition. Time-lapse imaging was started after a positively double-transfected cell for GFP–βII-spectrin and RFP–actin engaged with the fibronectin-coated coverslip. Images were acquired with a Hamamatsu Orca Flash 4.0 V2 sCMOS camera in custom-written LabView software. Post-image deskewing and deconvolution were performed using HHMI Janelia custom software and ten iterations of the Richardson–Lucy algorithm. Cells were tested for mycoplasma contamination.

## Supplementary Material



10.1242/joces.263473_sup1 Supplementary information
