# Supplementary material for: Dynamic mechanisms for membrane skeleton transitions
Source: J Cell Sci. 2025 Feb 28;138(4):JCS263473. doi: 10.1242/jcs.263473 (PMC11928055; doi:10.1242/jcs.263473)
Supplement: Supplementary information [file joces-138-263473-s1.pdf]

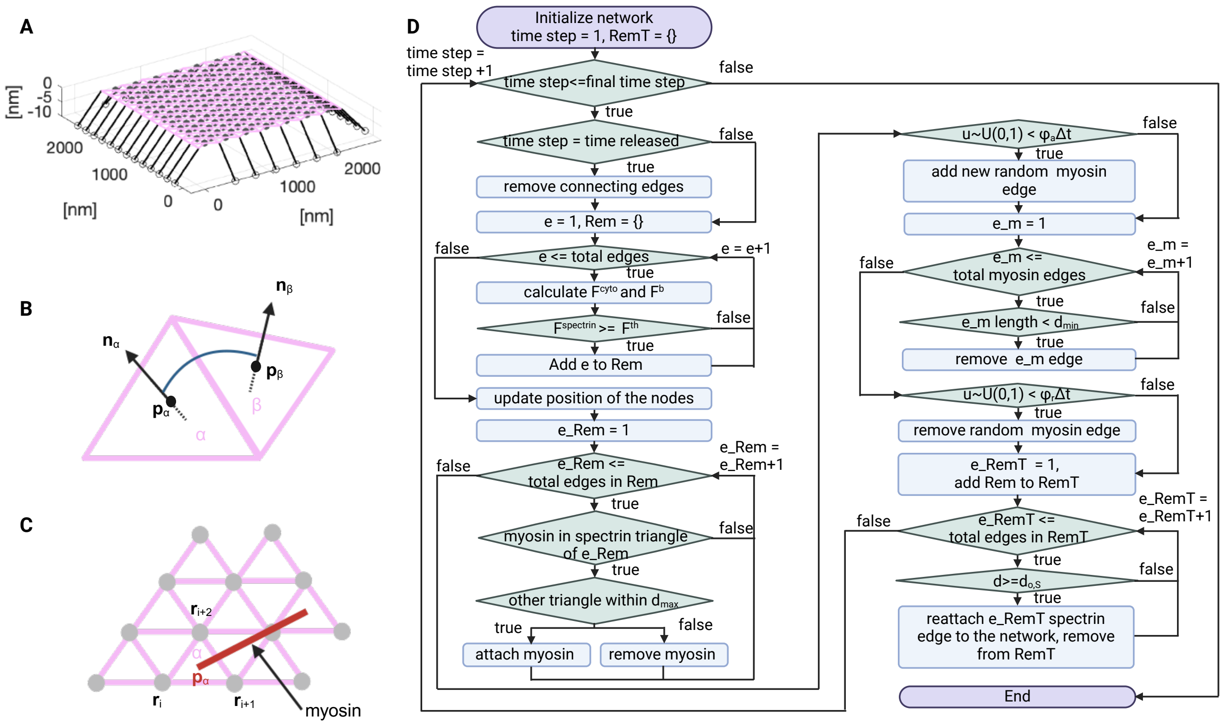

Fig. S1. Actin-spectrin meshwork simulation. A) 3D view of the initial configuration of the mesh. Pink and black lines correspond to spectrin and connector edges, respectively. Grey-filled circles are F-actin nodes and black empty circles represent focal adhesions. **B**) Schematic representation of the angle $\theta_{\alpha,\beta}$ formed by the α and β triangular faces of the meshwork. **C**) Myosin edges (red lines) endpoints are localized at the centers of the spectrin triangles (pink) at position $\mathbf{p}_\alpha = (\mathbf{r}_i + \mathbf{r}_{i+1} + \mathbf{r}_{i+2})/3$. The force generated by myosin is equally distributed between the F-actin nodes (gray) connecting the spectrin triangle. **D**) Flowchart of the simulation.

Journal of Cell Science • Supplementary information

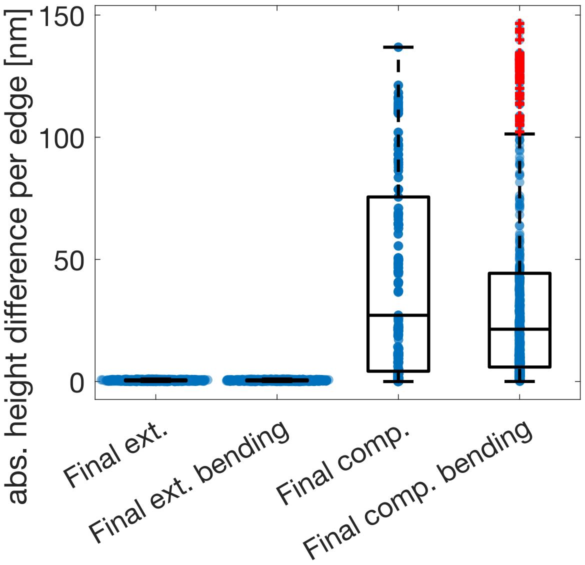

Fig. S2. Difference of edge height in the actin-spectrin meshwork under symmetrical extension and compression. We calculated the absolute value of the difference in height between the endpoints of each spectrin edge. Boxplot presenting the center (median), box (25th to 75th quartile, IQR), whiskers (±1.5× IQR), and asterisks (outliers). For each group n=509. The median values are 0.4721 (Final ext.), 0.4724 (Final ext. bending), 27.1247 (Final comp.), and 21.4208 (Final comp. bending). A two-sided Wilcoxon rank sum test rejects the null hypothesis that the medians of Final comp. and Final comp. bending are the same with a p-value of 0.0384. This figure is a supplement to Fig. 2.

Journal of Cell Science • Supplementary information

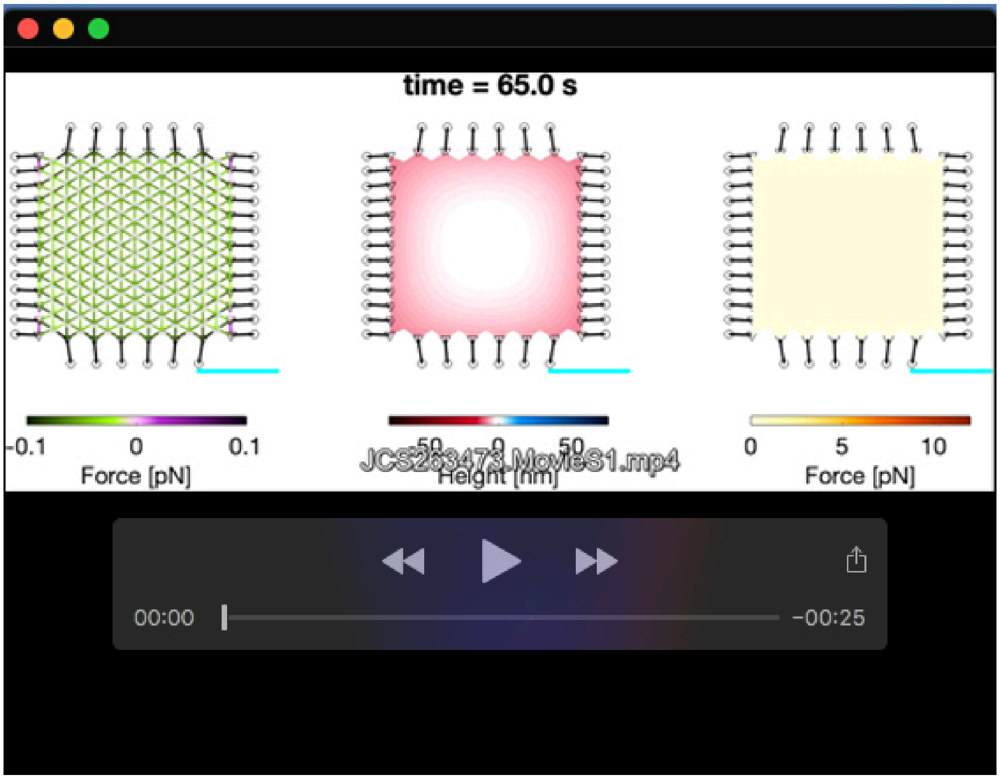

Movie 1. Actin-spectrin meshwork under symmetrical extension. Left: The edges corresponding to spectrin edges, color-coded for the force generated by their spring element. Center: Meshwork color-coded for the height of the F-actin nodes. Right: Meshwork color-coded for the magnitude of the force generated by the membrane. Movie corresponding to Fig. 2.

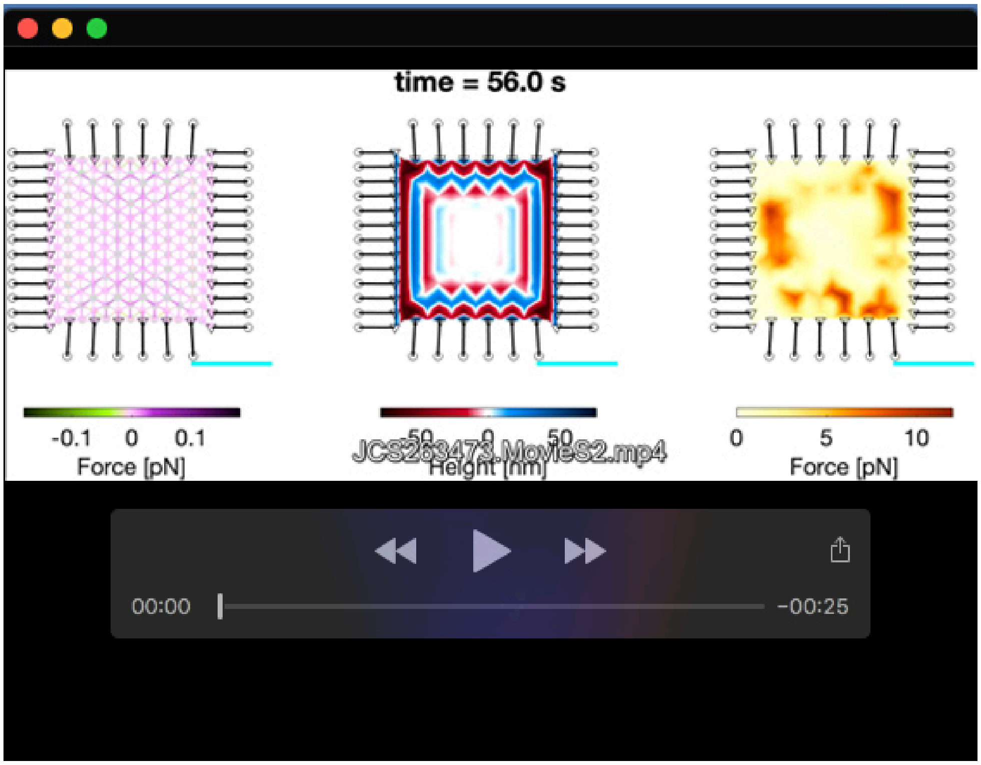

Movie 2. Actin-spectrin meshwork under symmetrical compression. Left: The edges corresponding to spectrin edges, color-coded for the force generated by their spring element. Center: Meshwork color-coded for the height of the F-actin nodes. Right: Meshwork color-coded for the magnitude of the force generated by the membrane. Movie corresponding to Fig. 2.

Journal of Cell Science • Supplementary information

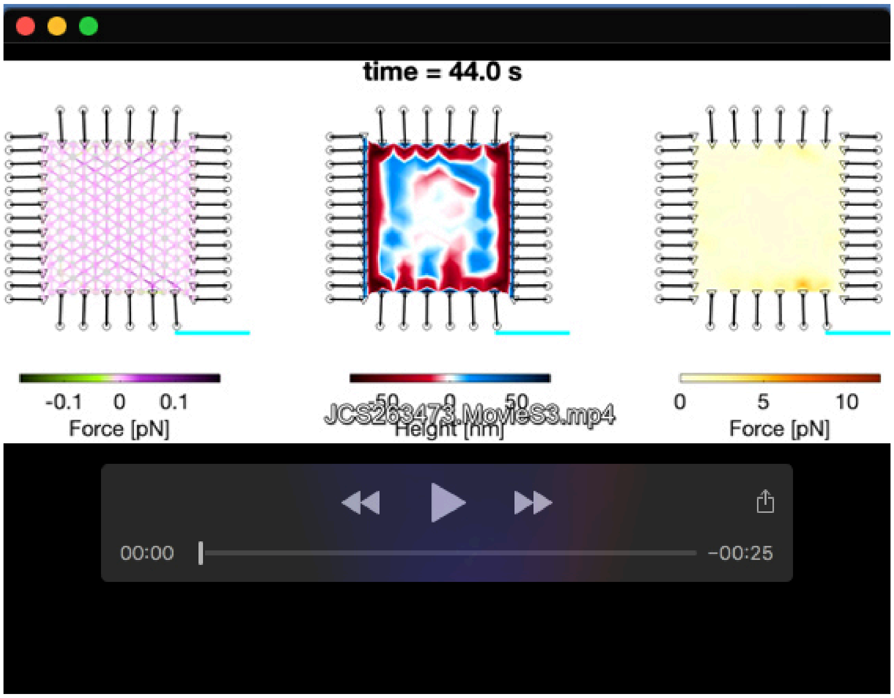

Movie 3. Actin-spectrin meshwork under symmetrical compression considering the membrane bending energy. Left: The edges corresponding to spectrin edges, color-coded for the force generated by their spring element. Center: Meshwork color-coded for the height of the F-actin nodes. Right: Meshwork color-coded for the magnitude of the force generated by the membrane. Movie corresponding to Fig. 2.

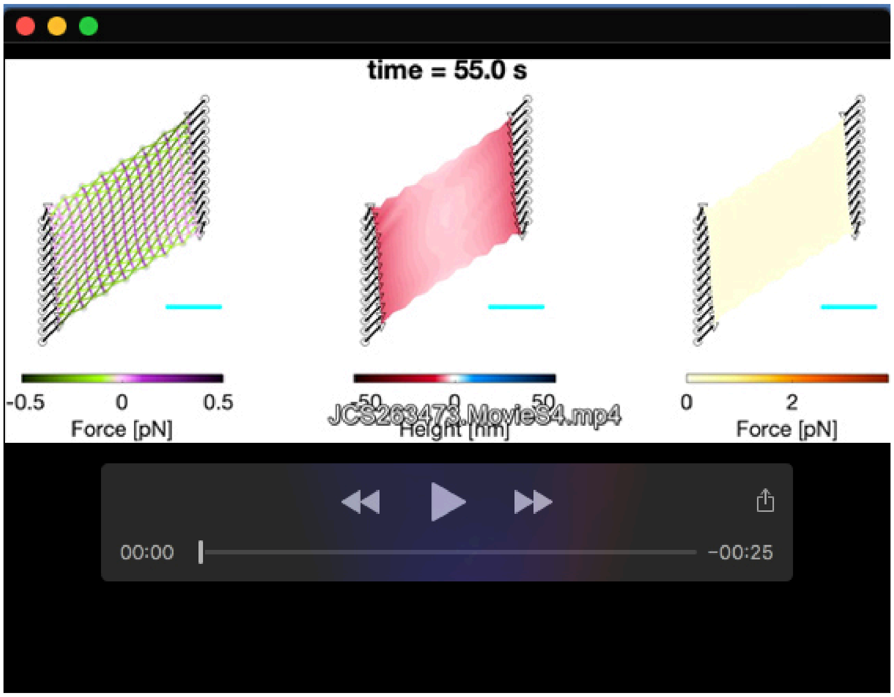

Movie 4. Actin-spectrin meshwork under shear stress. Left: The edges corresponding to spectrin edges, color-coded for the force generated by their spring element. Center: Meshwork color-coded for the height of the F-actin nodes. Right: Meshwork color-coded for the magnitude of the force generated by the membrane. Movie corresponding to Fig. 3.

Journal of Cell Science • Supplementary information

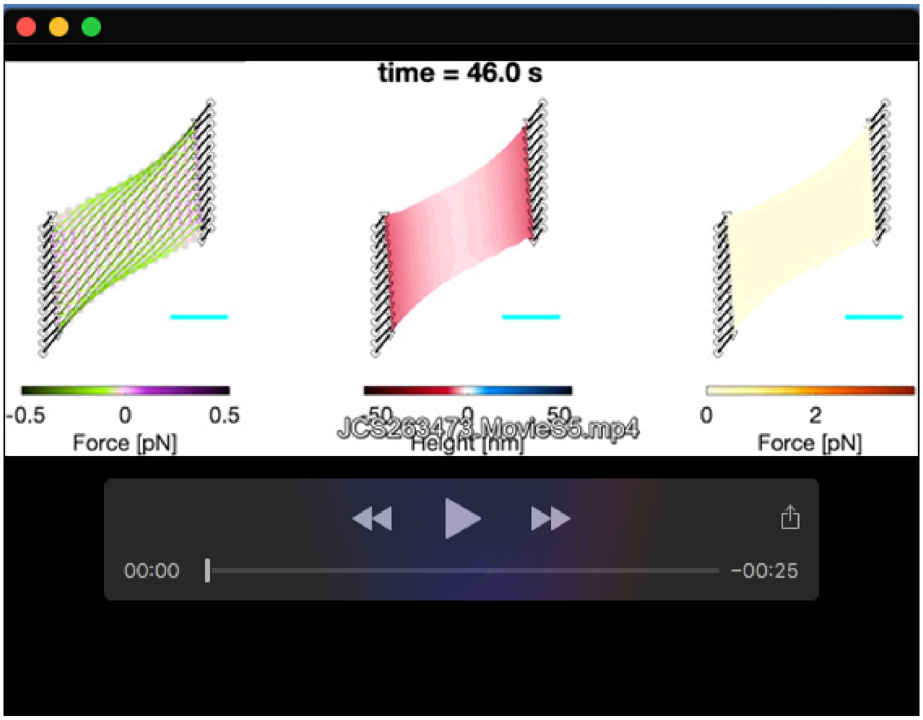

Movie 5. Actin-spectrin meshwork under shear stress with spectrin unbinding. Left: The edges corresponding to spectrin edges, color-coded for the force generated by their spring element. Center: Meshwork color-coded for the height of the F-actin nodes. Right: Meshwork color-coded for the magnitude of the force generated by the membrane. Movie corresponding to Fig. 3.

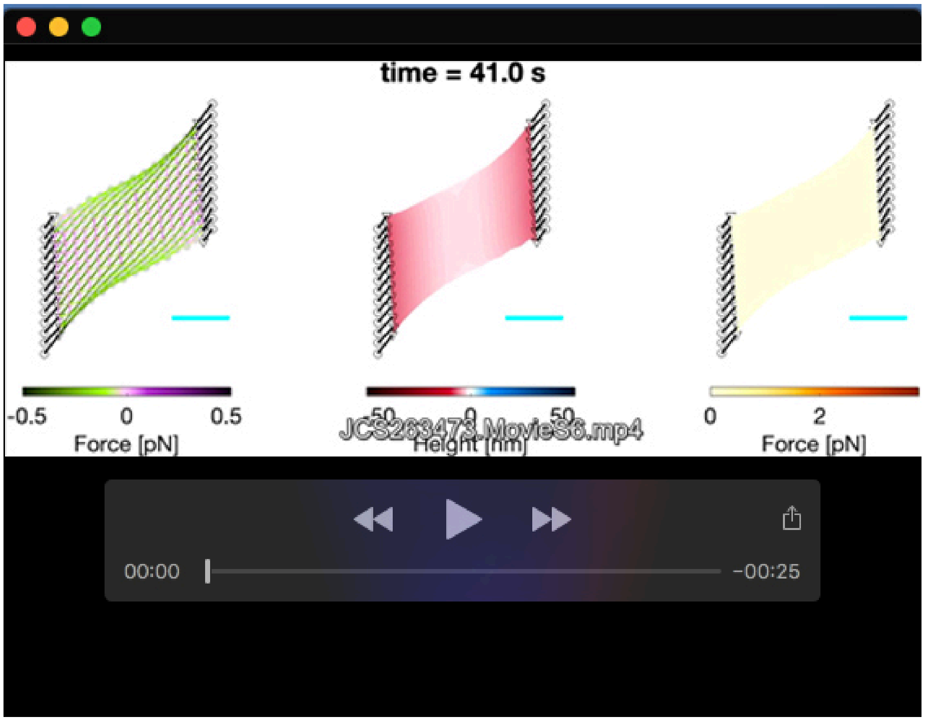

Movie 6. Actin-spectrin meshwork under shear stress with spectrin unbinding and rebinding. Left: The edges corresponding to spectrin edges, color-coded for the force generated by their spring element. Center: Meshwork color-coded for the height of the F-actin nodes. Right: Meshwork color-coded for the magnitude of the force generated by the membrane. Movie corresponding to Fig. 3.

Journal of Cell Science • Supplementary information

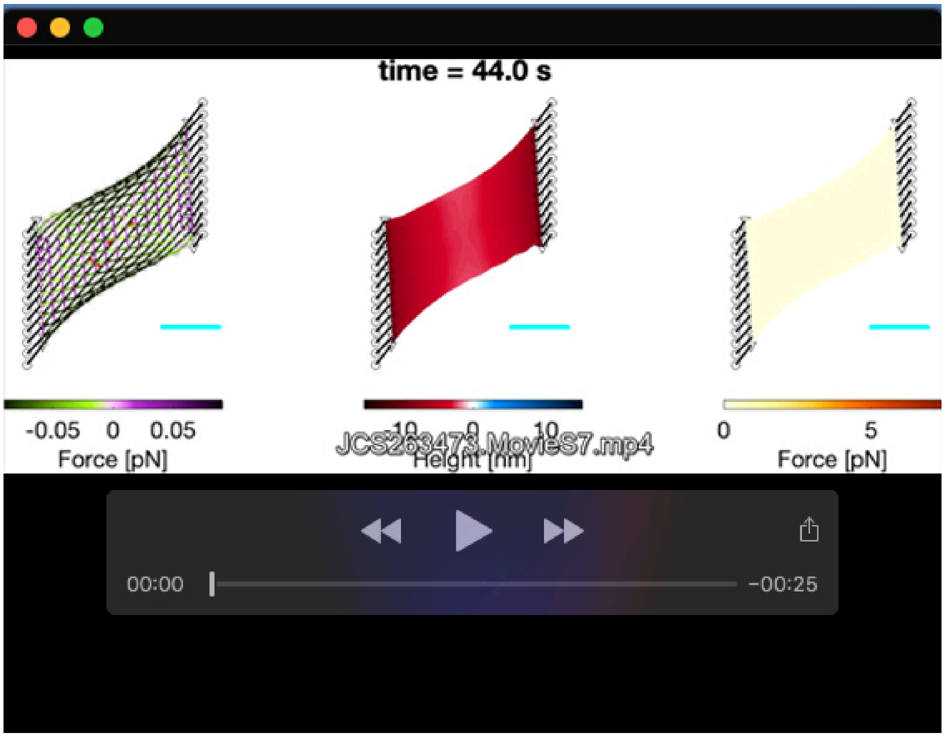

Movie 7. Myosin dynamics on an actin-spectrin meshwork under shear stress. Left: The edges corresponding to spectrin edges, color-coded for the force generated by their spring element. Center: Meshwork color-coded for the height of the F-actin nodes. Right: Meshwork color-coded for the magnitude of the force generated by the membrane. Movie corresponding to Fig. 4.

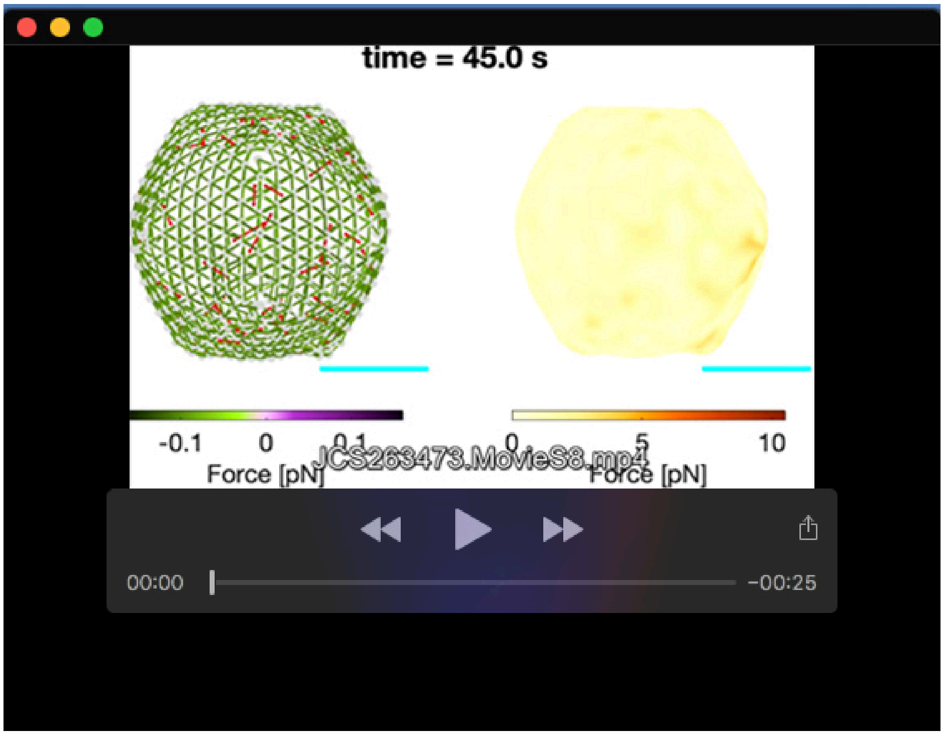

Movie 8. Actin-spectrin meshwork dynamics on a suspended cell. Left: The edges corresponding to spectrin edges, color-coded for the force generated by their spring element. Right: Meshwork color-coded for the magnitude of the force generated by the membrane. Movie corresponding to Fig. 6.

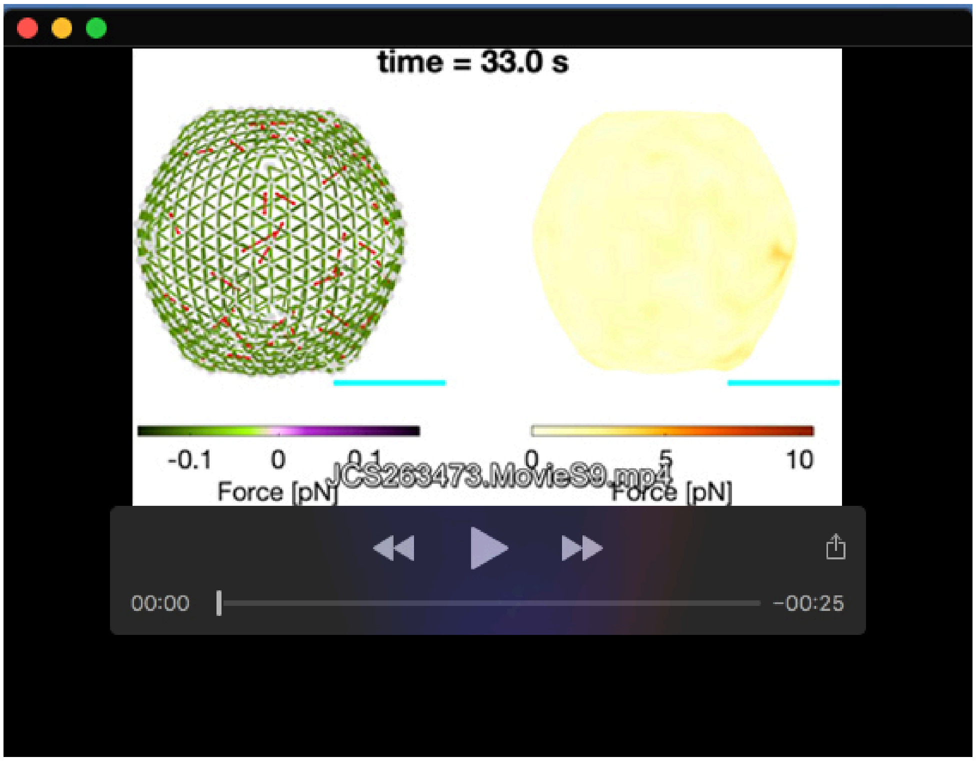

Movie 9. Actin-spectrin meshwork dynamics on a suspended cell with area constraint. Left: The edges corresponding to spectrin edges, color-coded for the force generated by their spring element. Right: Meshwork color-coded for the magnitude of the force generated by the membrane. Movie corresponding to Fig. 6.

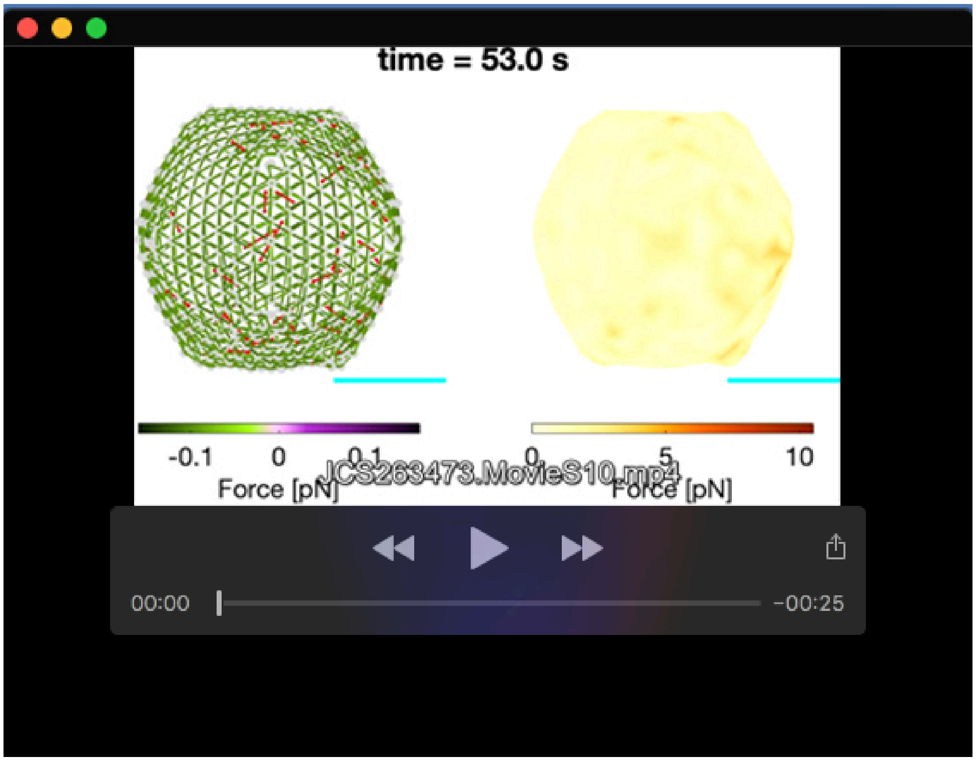

Movie 10. Actin-spectrin meshwork dynamics on a suspended cell with area constraint and volume exclusion. Left: The edges corresponding to spectrin edges, color-coded for the force generated by their spring element. Right: Meshwork color-coded for the magnitude of the force generated by the membrane. Movie corresponding to Fig. 6.

Journal of Cell Science • Supplementary information

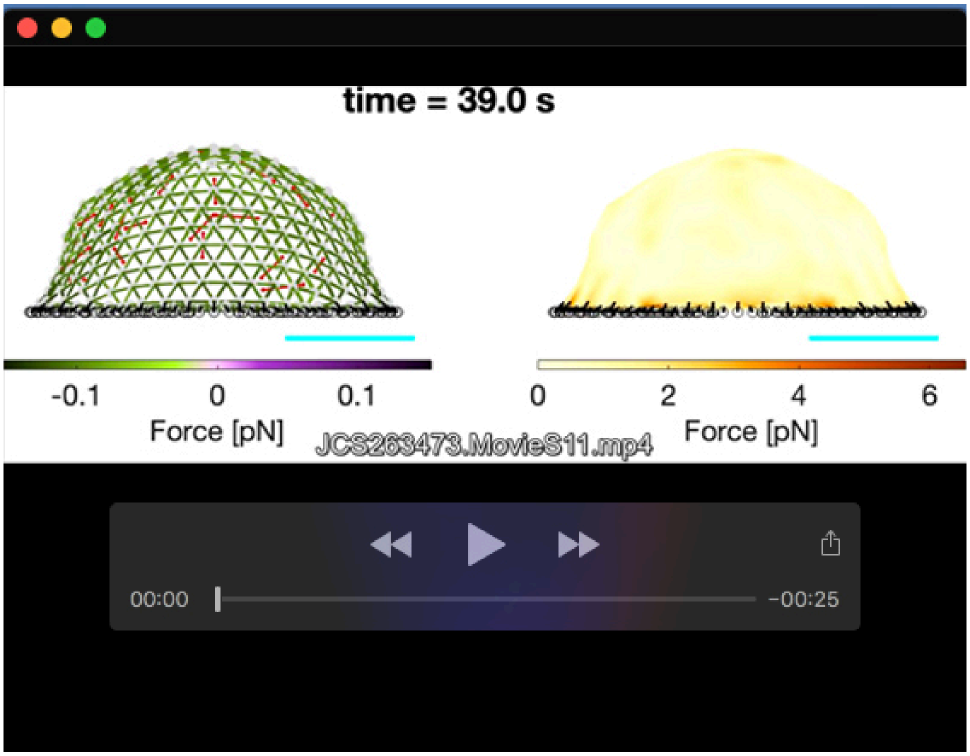

Movie 11. Actin-spectrin meshwork dynamics on an adhered cell. Left: The edges corresponding to spectrin edges, color-coded for the force generated by their spring element. Right: Meshwork color-coded for the magnitude of the force generated by the membrane. Movie corresponding to Fig. 7.

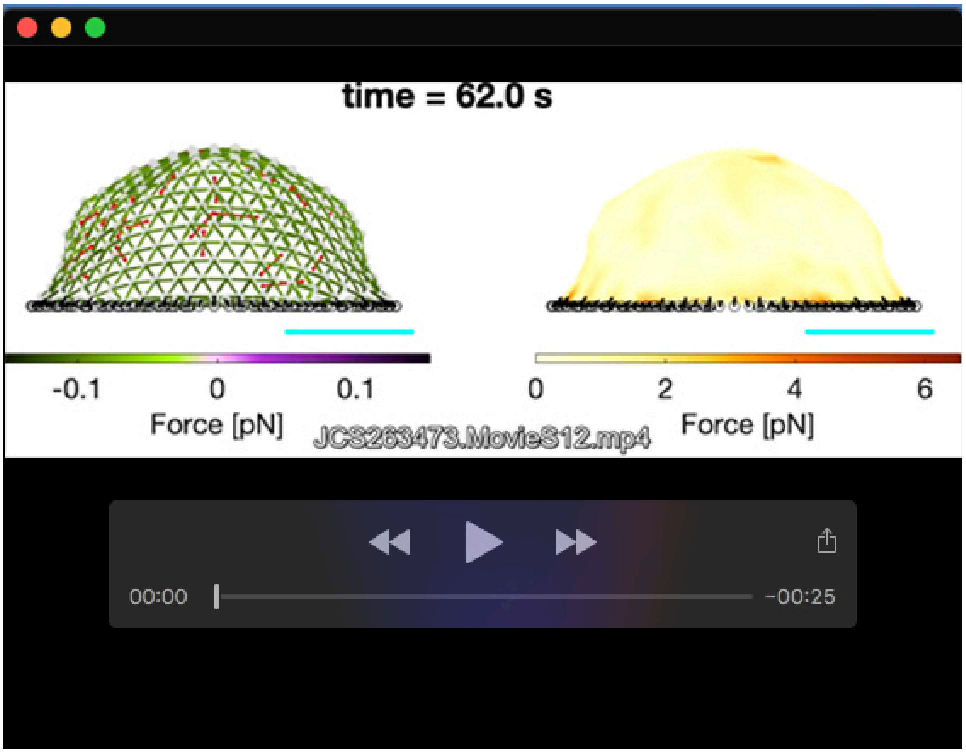

Movie 12. Actin-spectrin meshwork dynamics on an adhered cell with area constraint. Left: The edges corresponding to spectrin edges, color-coded for the force generated by their spring element. Right: Meshwork color-coded for the magnitude of the force generated by the membrane. Movie corresponding to Fig. 7.

Journal of Cell Science • Supplementary information

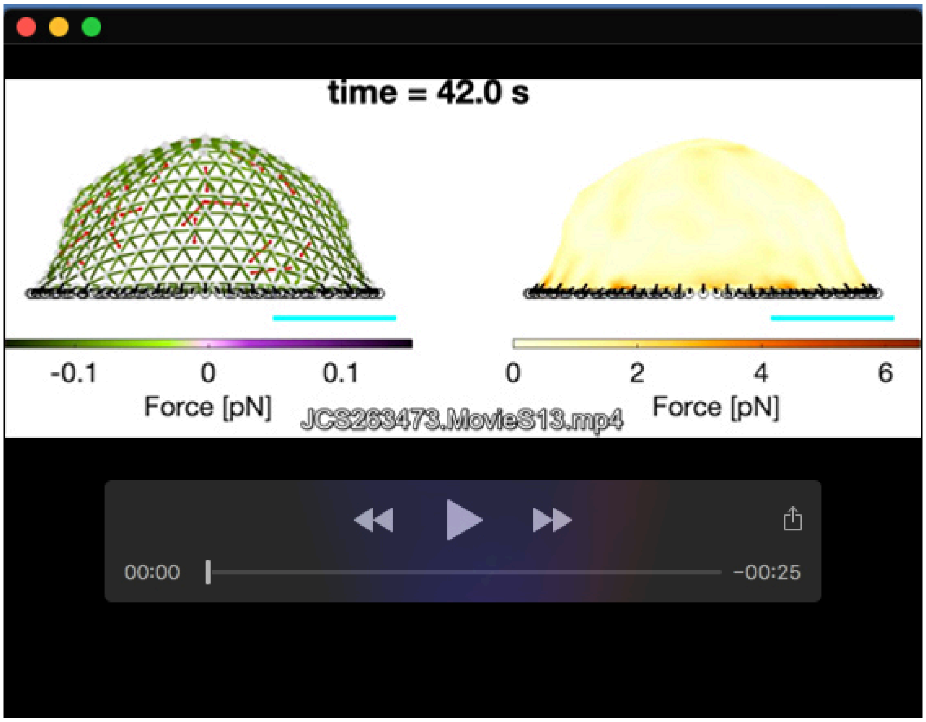

Movie 13. Actin-spectrin meshwork dynamics on an adhered cell with area constraint and volume exclusion. Left: The edges corresponding to spectrin edges, color-coded for the force generated by their spring element. Right: Meshwork color-coded for the magnitude of the force generated by the membrane. Movie corresponding to Fig. 7.

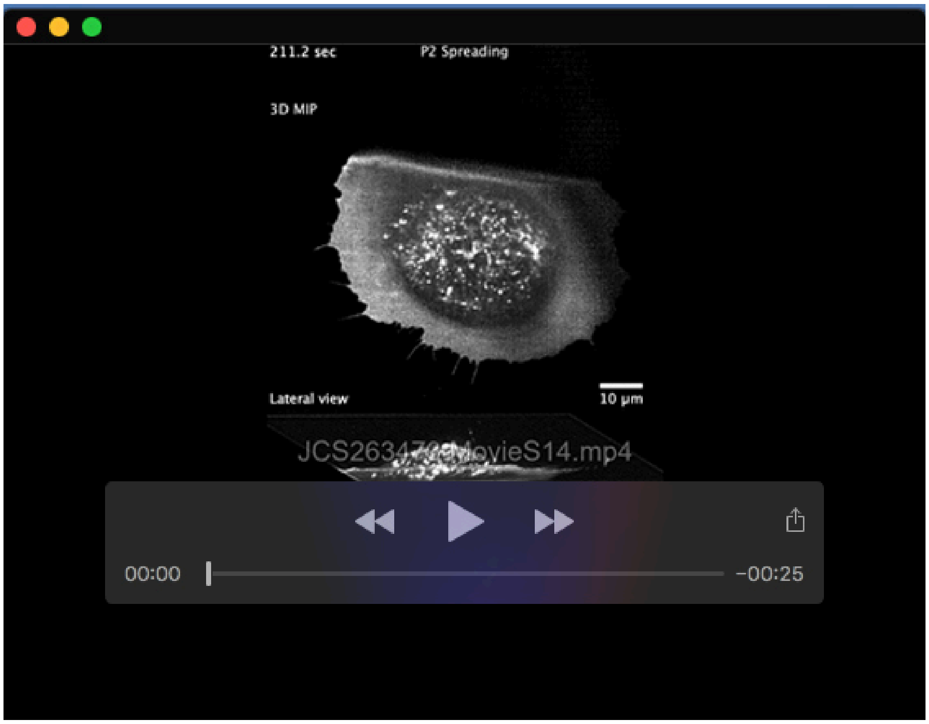

Movie 14. Cell spreading . Imaged by Lattice Light Sheet Microscopy in MEF transfected with the membrane reporter Scarlet-PM(Lck), scale bar: 10 µm. Movie corresponding to Fig. 7.

Journal of Cell Science • Supplementary information
