# Supplementary material for: Dynamic mechanisms for membrane skeleton transitions
Source: J Cell Sci. 2025 Feb 28;138(4):JCS263473. doi: 10.1242/jcs.263473 (PMC11928055; doi:10.1242/jcs.263473)
Supplement: Supplementary file 1 [file joces-138-263473_review_history.pdf]

Journal of Cell Science

Dynamic mechanisms for membrane skeleton transitions

Mayte Bonilla-Quintana, Andrea Ghisleni, Nils C. Gauthier, Padmini Rangamani
DOI: 10.1242/jcs.263473

Editor: Michael Way

Review timeline

Original submission:	7 August 2024
Editorial decision:	19 September 2024
First revision received:	30 December 2024
Accepted:	3 January 2025

Original submission

First decision letter

MS ID#: jcs.263473

MS TITLE: Dynamic mechanisms for membrane skeleton transitions

AUTHORS: Mayte Bonilla-Quintana; Andrea Ghisleni; Nils C. Gauthier; Padmini Rangamani

We have now reached a decision on the above manuscript.

To see the reviewers' reports and a copy of this decision letter, please go to:

As you will see, the reviewers raise a number of criticisms that prevent me from accepting the paper at this stage. They suggest, however, that a revised version might prove acceptable, if you can address their concerns. If you think that you can deal satisfactorily with the criticisms on revision, I would be pleased to see a revised manuscript. We would then return it to the reviewers.

Reviewer 1

Advance summary and potential significance to field

This is an interesting, novel computational study of cell cortex. The model is clear, logical, spectrin-centric. What I liked is linking of membrane-related energy with elastic deformations of the spectrin-actin network with myosin contractile action with network dynamics with adhesions to ECM with cytoplasm volume conservation. I have not seen this comprehensive attempt before, and here it is satisfying. Simulations predict and explain a number of effects, including:

- the membrane is not just supported by the skeleton, but has an active contribution to the stability of the cell structure.
- spectrin and myosin turnover are necessary for cortex relaxation.
- surface area constraint of the plasma membrane and volume restriction of the cytoplasm enhance the stability of the cortex.
- cell adhesions promotes cell shape stabilization.

All of this is very intuitive, but it is good to see the conceptually simple model giving these results. The paper is clearly written and is appropriate for JCS.

© 2025. Published by The Company of Biologists under the terms of the Creative Commons Attribution License (https://creativecommons.org/licenses/by/4.0/).

Comments for the author

I have two major critical comments and one minor one:

1) What is sorely missing in the paper is discussion of characteristic scales. The table of parameters in the methods part is absolutely not enough, not to mention that a lot of them are 'fitted' - fitted to what? What I expect from physicists on the team is to show the reader what combinations of dimensional parameters give characteristic dimensional scales and key non-dimensional parameters in the model. For example: after reading the paper, I have no clue about what is the hierarchy of time scales (for membrane folding? for cell spreading? etc), what mechanical and kinetic parameters determine these scales? Same about spatial scales.
Same about energy scales (i.e., how come membrane bending energy is relevant at all - multiple papers say that surface area and no flux at the membrane is all that is important; bending is negligible compared to the cortex - are they all wrong?). Etc.

2) the experimental part of the study - second half of fig 8 - is tiny and is an afterthought.
It does not strengthen the paper, and there are no quantitative information here. This part is unconvincing. i recommend to just remove it and simply discuss relevance to published experimental results.

Minor: fig 2 is not necessary in the main text, it can go to sup mat.

Reviewer 2

Advance summary and potential significance to field

The paper aims to model the spectrin network adsorbed to a membrane under conditions where local contractile forces, and external shearing and stretching forces are applied. This is a field that has a long history, mainly focused on the RBC cytoskeleton and shape, and more recently also in the context of other cell types.
The work here is interesting, but I find it is lacking in focus and clarity. The results are not clearly explained, and therefore its difficult to assess the contribution that is made here.

Comments for the author

1) On page 2 it says: "Interestingly, some experimental evidence suggests that the actin-spectrin meshwork in the RBC (7, 13, 18) and axons (19) is also dynamic."
The dynamics of the RBC spectrin network was shown by a variety of experimental and theoretical papers which are not cited at all and should be, such as:
Turlier, Hervé, et al. "Equilibrium physics breakdown reveals the active nature of red blood cell flickering." Nature physics 12.5 (2016): 513-519.
Park, YongKeun, et al. "Metabolic remodeling of the human red blood cell membrane." Proceedings of the National Academy of Sciences 107.4 (2010): 1289-1294.
Rodríguez-García, Ruddi, et al. "Direct cytoskeleton forces cause membrane softening in red blood cells." Biophysical journal 108.12 (2015): 2794-2806.
2) Fig.1C: Myosin-II is depicted here with only one end walking on the actin, while it normally forms multi-headed symmetric bundles that exert contractile forces by catching anti-parallel actin filaments and walking on both ends simultaneously. This is not what is shown here. Anyway, this geometry is not used for the rest of the paper, so no need to show it here.
3) Module 2.1.1: It is not clear if this gives a representation of the ATP-induced dynamics of the spectrin network ? it should be explained how this is a non-equilibrium process. The whole involvement of ATP in the spectrin dynamics is not mentioned, as well as the ATP-induced fluctuations and forces applied to the membrane (see refs in (1) above).
4) Page 6, it says: "Moreover, the function of the spectrin-ankyrin assembly is mostly associated with the organization of membrane proteins in domains (37)". These linkers also affect the stiffness of the spectrin springs:
Gov, Nir S. "Less is more: removing membrane attachments stiffens the RBC cytoskeleton." New Journal of Physics 9.11 (2007): 429.

© 2025. Published by The Company of Biologists under the terms of the Creative Commons Attribution License (https://creativecommons.org/licenses/by/4.0/).

Lai, Lipeng, et al. "Stiffening of red blood cells induced by cytoskeleton disorders: a joint theory-experiment study." Biophysical journal 109.11 (2015): 2287-2294.
5) Page 7, it says: "If one of the edges of the spectrin triangle is unbound, then the myosin edge tries to attach to a nearby triangle within a distance of dmax.", Why is the myosin-II contractility dependent on the existence of intact spectrin network ? surely the acto-myosin cortex can generate contractility at the membrane without the spectrin network. This is not clear.
6) Fig.4 shows the more interesting result in the paper, demonstrating how the spectrin turnover by unbinding/binding releases the formation of membrane folds due to the shearing. Again, its not clear if this process of unbinding/binding is ATP-dependent here.
7) Page 12, it says:"myosin edges generated a contractile force and were removed when they shrunk to a minimal length". Why is this condition realistic ? mysin motors can remain in a condensed state of motors and actin, forming asters (see for example: Gordon, Daniel, et al. "Hierarchical self-organization of cytoskeletal active networks." Physical biology 9.2 (2012): 026005.)
8) Fig.5: Panels G,I show that the myosin has no effect on the spectrin network, right ? so why does the text say otherwise ?
9) Fig.6A,B: something happens at time~110s, but its not explained in the caption
10) Page 17, it says: "However, during the first few seconds of the simulation, the edges with smaller lengths than the resting length were removed". Why ? spectrin filaments can extend into the cytosplasm and therefore their end nodes can easily shrink below the rest length.
11) Page 17: "spectrin edges unbound" -> unbound spectrin edges, "spectrin edges attached was"-> attached spectrin edges was.
12) Page 17, it says: "While the actin-spectrin meshwork allows cells in suspension to deform and bear different stresses, the membrane surface area constraint and volume exclusion of the cytoplasm guarantee shape integrity. This complements the accepted function of the spectrin skeleton in giving mechanical support to the membrane (5) and hints at a feedback mechanism between the membrane and the skeleton." But both of these, the area conservation and excluded volume, do not depend on the spectrin, so how adding them shows that the spectrin is contributing to maintaining a stable shape ?
13) Fig.7F shows that changes are still significant at the final times, so these are not at steady-state yet.
14) Page 19, it says:"J). These correlative observations closely resemble the series of events captured by our model, suggesting that the enhanced stability of the adherent meshwork is important for cell function." Its not clear what observations exactly correspond to what the model predicts, ie which results in panels A-F correspond to the experimental observations. Why dont they plot the cell adhered area and height as in the experiment for comparison ? The claimed agreement is not clearly shown.
15) Fig.8: Values and units are missing along the y-axis in H,J.
16) Page 21: "as on a recent model"-> as in a recent model
17) Table 1: the bending modulus of (400kBT) is much larger than values measured in previous studies (see refs in (1)).

<u>Reviewer 3</u>

Advance summary and potential significance to field

This manuscript develops a computational biomechanics model for the deformations of a cell's actin-spectrin skeleton coupled to the cell membrane. The model incorporates key mechanical contributions to this cellular system: the bending elasticity of the membrane, surface and volume constraints from the membrane and cytoplasm, as well as the turnover of myosin motors and spectrin structures, and shows that these factors contribute to the stability of cell shape. While the modeling is thorough and can potentially inform future experiments on this important biophysical system, I have reservations for publication in current form detailed below. These mainly center on some modeling choices that are not made clear, and the lack of any quantitative comparison with experiments (or even a discussion of what testable predictions the model makes that moves understanding of cell biological mechanisms forward).

Comments for the author

© 2025. Published by The Company of Biologists under the terms of the Creative Commons Attribution License (https://creativecommons.org/licenses/by/4.0/).

Major

1. Erythrocytes (red blood cells) are the prototypical cells that contain a 2D polygonal lattice structure of actin and spectrin. This seems to inspire the geometry of the spring network used in this model. It is not made clear if the model is specific to erythrocytes, or do the authors have other cells in mind as well? In the former case, the abstract and Discussion should specifically mention red blood cells. Some comments on how the model can be generalized to spectrin-actin skeleton in neurons and fibroblasts would also broaden the scope of the work.

2. As mentioned in the Discussion, other modeling approaches describe spectrin as worm like chains. What justifies the neglect of bending and buckling of the spectrin? Maybe the authors can provide some estimate of the persistence length/bending elasticity of spectrin that justifies this choice. Further, it is well known that bending stiffness has an important stabilizing effect on disordered elastic networks (see for example, Broedersz et al. Nat. Phys. 7, 983-988 (2011); Das et al. PLOS ONE 7, e35939 (2012)) This effect may be important if the authors go away from their fully connected polygonal lattice geometry, and this regime may also occur in the current model when spectrin springs are removed. Have they measured the mean connectivity (coordination number) of their network in time? Do the simulated networks remains above the critical coordination number given by Maxwell's count?

3. What is the experimental evidence that justifies the removal criterion of spectrin edges under compression? This should be discussed at the outset. Also, this choice has an interesting analogy with models that include buckling of fibers, since a buckled bond provides negligible resistance to compression.

4. It is known that myosin binding to actin involves catch bonds, i.e. a force dependent on and off rate. Can the authors include this in their model to see how the results change?

5. Fig. 3 B shows that the membrane is still high when membrane bending is included. The plot in 3B for meshwork height with bending included also shows highly blue and red regions that have high positive and negative heights. This is apparently contradictory to the authors claim in the text that bending reduces height fluctuations . The claim is not supported by the plot in 3E. The difference between the box plot for the case of compression and compression with bending are not apparent.

6. What observations in the cell spreading experiment can be quantitatively explained by this model? Can the model be used to generate cell area vs time plots like those shown in Figs. 8H and 8J? What are the testable predictions of the model that may be checked in future experiments?

7. Can the model say anything about the contributions of the spectrin skeleton to the old problem of flickering of the membrane of RBCs? An important reference on this topic: Turlier et al. Nature Phys 12, 513-519 (2016).

Minor

1. Line 261, the figure referenced should be 2A and not 2B.
2. Line 288: F-actin z-node position may have some extra points inside the box plot for the case of compression with bending but it also has a higher range in both positive and negative side - while the median is about the same. We suggest that the actual values of the median be reported and their difference be checked for statistical significance.
3. Fig. 4G has two green colored dashed lines. What is the difference between them?
4. Fig 4D: When meshwork is released (in both unbinding and unbinding+rebinding cases), it should have equal amounts of tensile and compressive forces - as mandated by force balance. However, from plots in A, it seems that most of the spectrin edges are compressed and apply a positive force. This deviation from rest-length is however pretty small as per 4D. It would be more instructive to apply a threshold for the color of edges so that the edges appear to be at rest-length then or an explain the deviation from rest-length.
5. In Fig. 4D, a horizontal line at y=180 nm would be useful.
6. Line 333: In the model, spectrin rebinding happens when the distance between two F-actin nodes becomes equal to rest-length. In practice, does this mean that there is a small threshold around the 180 nm rest length at which the spring reappears?

© 2025. Published by The Company of Biologists under the terms of the Creative Commons Attribution License (https://creativecommons.org/licenses/by/4.0/).

7. Fig. 6C: the percentage of attached edges seems to be slightly above the value for rr/5 and 5rr. Although two values greater than rr have been used to establish this convincingly (5rr and 10rr), it may make this work better with another value smaller than rr is used as well apart from rr/5. Moreover, since 30 simulations have been used, how statistically significant is the difference between rr and rr/5?
8. 6E: the box plot of spectrin edge lengths does not convincingly establish why rr is a better parameter than other values, specifically, rr/5. 6F: it is also not abundantly clear form the height data why rr is a better parameter than rr/5, as claimed in line 387. Lines 383-385 should also mention the corresponding IQR values for rr/5 and establish how this is significantly worse than those for corresponding values of rr.
9. Line 421: Myosin removal is a purely stochastic event so why should the change in force balance lead to the subsequent removal of myosin?
10. Fig. 3B: Why does addition of bending membrane force break the left-right and top-bottom symmetry when compared with the case of isotropic compression without bending? Even with bending force, the symmetry should be maintained.
11. Fig. 5G: There does not seem to be a significant difference between the two cases without myosin (dashed lines) and with myosin (solid lines). This does not support the statement at line 356.

First revision

<u>Author response to reviewers' comments</u>

Dear Handling Editor Michael Way and reviewers:

We thank you for your comments on our manuscript, which has been edited to comply with the formatting guidelines. We have moved Figure 2 to the Supplementary Information to adhere to the allowed number of figures. The changes to the text addressing the reviewers' comments are highlighted in yellow. Below is our point-by-point response. Note that we used italicized font to quote the reviewers; our responses are in blue.

Thank you for your consideration and we look forward to a positive response,
Padmini Rangamani and Nils Gauthier

Padmini Rangamani
Professor
Department of Pharmacology, School of Medicine
Department of Mechanical and Aerospace Engineering, Jacobs School of Engineering
University of California San Diego

Nils Gauthier
Principal Investigator
Mechano-Oncology
Lab
Institute FIRC of Molecular Oncology (IFOM), Italy.
—---

Note that Figure 2 is now Supplementary Figure 1. Therefore, the order of Figures has changed accordingly.

Reviewer 1:

SUMMARY OF THE ADVANCE MADE IN THIS PAPER AND ITS POTENTIAL SIGNIFICANCE TO THE FIELD

This is an interesting, novel computational study of cell cortex. The model is clear, logical,

© 2025. Published by The Company of Biologists under the terms of the Creative Commons Attribution License (https://creativecommons.org/licenses/by/4.0/).

spectrin-centric. What I liked is linking of membrane-related energy with elastic deformations of the spectrin-actin network with myosin contractile action with network dynamics with adhesions to ECM with cytoplasm volume conservation. I have not seen this comprehensive attempt before, and here it is satisfying. Simulations predict and explain a number of effects, including:
- the membrane is not just supported by the skeleton, but has an active contribution to the stability of the cell structure.
- spectrin and myosin turnover are necessary for cortex relaxation.
- surface area constraint of the plasma membrane and volume restriction of the cytoplasm enhance the stability of the cortex.
- cell adhesions promotes cell shape stabilization.

All of this is very intuitive, but it is good to see the conceptually simple model giving these results. The paper is clearly written and is appropriate for JCS.
We appreciate the kind comments of Reviewer #1.

SUGGESTIONS TO AUTHORS

I have two major critical comments and one minor one:

R1.1) What is sorely missing in the paper is discussion of characteristic scales. The table of parameters in the methods part is absolutely not enough, not to mention that a lot of them are 'fitted' - fitted to what? What I expect from physicists on the team is to show the reader what combinations of dimensional parameters give characteristic dimensional scales and key non-dimensional parameters in the model. For example: after reading the paper, I have no clue about what is the hierarchy of time scales (for membrane folding? for cell spreading? etc), what mechanical and kinetic parameters determine these scales? Same about spatial scales. Same about energy scales (i.e., how come membrane bending energy is relevant at all - multiple papers say that surface area and no flux at the membrane is all that is important; bending is negligible compared to the cortex - are they all wrong?). Etc.
We agree with the reviewer that a discussion of the characteristic scales would give the reader a better understanding of how the mechanical parameters determine time and spatial scales. However, the mechanical parameters for spectrin and myosin were taken from Ghisleni, et al. 2024, where they were fitted to replicate the experiments qualitatively. Thus, any resulting temporal and spatial scales in the model are influenced by these fitted parameters and any conclusion regarding the scales could be inaccurate. We acknowledge that this is an important area of research and this is something our team aims to do in the future.
To better explain our choices for the parameters, we have added the “Parameter fit” subsection to the “Materials and Methods” section (lines 468-482). In short, we set the spatial parameters to resemble experiments and fit the mechanical parameters. We have also added this to the discussion section (lines 431-434): “The parameters related to spectrin and myosin mechanical properties were taken from (Ghisleni, et al., 2024). These model parameters were fitted to replicate experimental observations instead of taking values from the literature, which differ depending on cell type and experimental settings. Future efforts could change these fitted mechanical parameters to the experimental values for different types of cells and thereby, investigate whether the characteristic scales change.”

R1.2) the experimental part of the study - second half of fig 8 - is tiny and is an afterthought. It does not strengthen the paper, and there are no quantitative information here. This part is unconvincing. i recommend to just remove it and simply discuss relevance to published experimental results.
We appreciate Reviewer 1’s viewpoint on this figure. However, reviewers 2 and 3 asked us to compare the data in Fig.8 (now Fig.7) with the model. Therefore, we have decided to keep it by adding quantitative information and comparing it with the simulation results (see R2.14 and R3.6). We hope that the reviewer sees this as an acceptable compromise.

R1.3) Minor: fig 2 is not necessary in the main text, it can go to sup mat.
We agree with the reviewer and moved the figure to the Supplementary Material.

© 2025. Published by The Company of Biologists under the terms of the Creative Commons Attribution License (https://creativecommons.org/licenses/by/4.0/).

Reviewer 2:

SUMMARY OF THE ADVANCE MADE IN THIS PAPER AND ITS POTENTIAL SIGNIFICANCE TO THE FIELD
The paper aims to model the spectrin network adsorbed to a membrane under conditions where local contractile forces, and external shearing and stretching forces are applied. This is a field that has a long history, mainly focused on the RBC cytoskeleton and shape, and more recently also in the context of other cell types.
The work here is interesting, but I find it is lacking in focus and clarity. The results are not clearly explained, and therefore its difficult to assess the contribution that is made here.
We have revised our manuscript to make many of the points clearer. As reviewer #2 stated, the spectrin network has been mostly studied in RBC. In this work, we aimed to give a more general vision of this spectrin network beyond the RBC and focus on the underlying mechanical features. We hope that our revised manuscript provides more clarity on our findings.

SUGGESTIONS TO AUTHORS

R2.1) On page 2 it says: "Interestingly, some experimental evidence suggests that the actin-spectrin meshwork in the RBC (7, 13, 18) and axons (19) is also dynamic."
The dynamics of the RBC spectrin network was shown by a variety of experimental and theoretical papers which are not cited at all and should be, such as:
Turlier, Hervé, et al. "Equilibrium physics breakdown reveals the active nature of red blood cell flickering." Nature physics 12.5 (2016): 513-519.
Park, YongKeun, et al. "Metabolic remodeling of the human red blood cell membrane." Proceedings of the National Academy of Sciences 107.4 (2010): 1289-1294.
Rodríguez-García, Ruddi, et al. "Direct cytoskeleton forces cause membrane softening in red blood cells." Biophysical journal 108.12 (2015): 2794-2806.
We thank the reviewer for pointing us to these studies, which we have added to the paper.

R2.2) Fig.1C: Myosin-II is depicted here with only one end walking on the actin, while it normally forms multi-headed symmetric bundles that exert contractile forces by catching anti-parallel actin filaments and walking on both ends simultaneously. This is not what is shown here. Anyway, this geometry is not used for the rest of the paper, so no need to show it here.
We thank the reviewer for this observation. We have updated Figure 1C to show myosin bipolar filaments walking on both ends.

R2.3) Module 2.1.1: It is not clear if this gives a representation of the ATP-induced dynamics of the spectrin network ? it should be explained how this is a non-equilibrium process. The whole involvement of ATP in the spectrin dynamics is not mentioned, as well as the ATP-induced fluctuations and forces applied to the membrane (see refs in (1) above).
As mentioned throughout the paper, we chose to represent the spectrin skeleton as a network of Hookean springs that lacks molecular detail. Therefore, we excluded the ATP-induced dynamics from the model, like ATP-dependent phosphorylation of the 4.1 protein or ATP-dependent myosin workload. Moreover, to our knowledge, such ATP-induced flickering has been observed only in RBC. We acknowledge this weakness in our model and point out the experimental and theoretical work describing the flickering motions in human red blood cells. We have added the following paragraph before Module 1 (lines 95-96): “Because our focus is on mechanical responses, we do not explicitly model ATP-driven processes that could be involved (Rodriguez-Garcia, et al., 2015, Park, et al., 2010, Turlier, et al.,2016).”
We also added the following paragraph to the discussion (lines 398-400):
“In RBC, experimental and theoretical efforts have described how the ATP-driven flickering of the membrane can be related to spectrin skeleton remodeling (Rodriguez-Garcia, et al., 2015, Park, et al., 2010, Turlier, et al., 2016), thereby, connecting RBC deformation to spectrin morphology.”

R2.4) Page 6, it says: "Moreover, the function of the spectrin-ankyrin assembly is mostly associated with the organization of membrane proteins in domains (37)". These linkers also affect the stiffness of the spectrin springs:
Gov, Nir S. "Less is more: removing membrane attachments stiffens the RBC cytoskeleton." New Journal of Physics 9.11 (2007): 429.
Lai, Lipeng, et al. "Stiffening of red blood cells induced by cytoskeleton disorders: a joint

© 2025. Published by The Company of Biologists under the terms of the Creative Commons Attribution License (https://creativecommons.org/licenses/by/4.0/).

theory-experiment study." Biophysical journal 109.11 (2015): 2287-2294.
Thank you for sharing these references. We have added the following sentence to the manuscript (lines 140-142): "Previous work modeling a mid-point attachment (ankyrin) on the edges corresponding to spectrin bundles, which are represented as two springs in series, showed that the network stiffness can lower when the attachments are removed (Gov, 2007). We assumed that this effect in the network is comparable to removing the spectrin edges in our model.".

R2.5) Page 7, it says: "If one of the edges of the spectrin triangle is unbound, then the myosin edge tries to attach to a nearby triangle within a distance of dmax.", Why is the myosin-II contractility dependent on the existence of intact spectrin network ? surely the acto-myosin cortex can generate contractility at the membrane without the spectrin network. This is not clear.
In our previous work (Ghisleni, et al., 2024), we chose to place the myosin edges in the center of the triangles because there was no colocalization between cortical myosin puncta and spectrin. Moreover, we observed a pulsatile behavior in myosin in spectrin-rich zones. Thus, we proposed that the force generated by myosin is distributed between the three actin nodes forming the spectrin triangle. In preliminary simulations, we observed that keeping the force distribution between the nodes forming the spectrin triangle when a spectrin edge was removed had a huge effect on the spectrin network geometry. We speculated that the actin nodes missing a spectrin edge (and the corresponding spring force) were very sensitive to the myosin force, leading to a change in position inside neighboring triangles. Furthermore, myosin edges did not show a pulsative behavior. To avoid huge effects in the geometry and obtain changes in the position of the myosin punta resembling the pulsative behavior, we propose myosin unbinding if the spectrin triangle was disrupted.
We agree with the reviewer that myosin could generate contractility at the membrane without spectrin and we have made this point clear in the text by adding the following information (lines 175-177): "We chose this rule because it avoids a sudden change in position of the actin nodes attached to the missing spectrin edge and allows dynamic changes of myosin position resembling the pulsative behavior seen in experiments (Ghisleni, et al., 2024). However, myosin contractility at the membrane is possible without a spectrin network."

R2.6) Fig.4 shows the more interesting result in the paper, demonstrating how the spectrin turnover by unbinding/binding releases the formation of membrane folds due to the shearing. Again, its not clear if this process of unbinding/binding is ATP-dependent here.
Unbinding of spectrin can be ATP-driven, as pointed out in the suggested references in point R2.1. As mentioned in our response to point R2.3, we did not model ATP-dependent mechanisms. Rather, the unbinding was dependent on the force generated by the spring element and the rebinding by the distance between two actin nodes previously connected by a spectrin edge (see Module 1: Mechanics of actin-spectrin meshwork for details).

R2.7) Page 12, it says:"myosin edges generated a contractile force and were removed when they shrunk to a minimal length". Why is this condition realistic ? mysin motors can remain in a condensed state of motors and actin, forming asters (see for example: Gordon, Daniel, et al. "Hierarchical self-organization of cytoskeletal active networks." Physical biology 9.2 (2012): 026005.)
Our modeling decisions for myosin are based on our previous work on spectrin clusters in fibroblasts (Ghisleni, et al., 2024, see response to R1.2). There, short-lived myosin pulses are observed and no increase in concentration of myosin walking on actin filaments, as needed for aster formation. We have included more details about our modeling choices in Module 4: Myosin dynamics (lines 178-180): "Note that if myosin is allowed to shrink indefinitely ($d \to 0$), then its force grows unbound ($F^{cable,M}_{\alpha} \to \infty$). Moreover, removing myosin prevents their concentration over time in line with experimental observations of short-lived myosin pulses, as shown in (Ghisleni et al., 2024)." We have also addressed why we did not include aster formation in our model (see R3.4).

R2.8) Fig.5: Panels G,I show that the myosin has no effect on the spectrin network, right ? so why does the text say otherwise ?
We have run one sample t-test to compare the end value of the simulations without myosin to the mean of the end value of the simulations with myosin and demonstrate

© 2025. Published by The Company of Biologists under the terms of the Creative Commons Attribution License (https://creativecommons.org/licenses/by/4.0/).

that the re-bound edges and percentage of attached edges are significantly different (p-value 5.2923×10^{-5} and 0.0026, respectively) in line with the text. We have added these values to the legend of the corresponding Figure.

R2.9) Fig.6A,B: something happens at time~110s, but its not explained in the caption
We thank the reviewer for the observation. We have added the following description to the legend: “Black vertical line denotes the time of network release from shear stress.”

R2.10) Page 17, it says: "However, during the first few seconds of the simulation, the edges with smaller lengths than the resting length were removed". Why ? spectrin filaments can extend into the cytosplasm and therefore their end nodes can easily shrink below the rest length.

We think the reviewer misunderstood the meaning of "were removed", which is not done deliberately but as a consequence of the spectrin edge being shorter than the resting length and generating a shrinking force above a threshold (F^{th}) at the beginning of the simulation. Note that for the cell in suspension and adhered cell, we used the same dynamics for spectrin as in the actin-spectrin meshwork patches. Therefore, the spectrin edges that generate a shrinking force above a threshold (F^{th}) are removed from the simulation. Since the initial configuration of the sphere has spectrin edges that are shorter than the resting length and generate a high shrinking force, they are quickly removed from the network. We have edited the sentence to include these details (lines 315-317): “The spectrin edges with an initial length smaller than the resting length, generated a high shrinking force, in some cases above the threshold F^{th}. Therefore, they were removed shortly after the start of the simulation (blue line, Fig. 6 D,E).”

R2.11) Page 17: "spectrin edges unbound" -> unbound spectrin edges, "spectrin edges attached was"-> attached spectrin edges was.

Modifications were done accordingly.

R2.12) Page 17, it says: "While the actin-spectrin meshwork allows cells in suspension to deform and bear different stresses, the membrane surface area constraint and volume exclusion of the cytoplasm guarantee shape integrity. This complements the accepted function of the spectrin skeleton in giving mechanical support to the membrane (5) and hints at a feedback mechanism between the membrane and the skeleton." But both of these, the area conservation and excluded volume, do not depend on the spectrin, so how adding them shows that the spectrin is contributing to maintaining a stable shape ?

We decided to investigate the relationship between spectrin dynamics and cell size following previous experimental observations of spectrin KO clones showing reduced cell size (Ghisleni et al., 2020).
Our model assumes a force balance between the elements of the membrane skeleton (Eq. 9). Therefore, adding membrane surface area constraint and volume exclusion of the cytoplasm to the model affects this force balance and we showed that it enhances the shape integrity. In the sentence, we wanted to emphasize that the contribution of spectrin is not only passively supporting the membrane but actively interacting with it. We have edited the sentence to avoid confusion (lines 341-347): “While the actin-spectrin meshwork allows cells in suspension to deform and bear different stresses, the membrane surface area constraint and volume exclusion of the cytoplasm guarantee shape integrity. Since the evolution of the actin nodes position dictating the cell shape is governed by the force balance between the membrane skeleton forces (Eq. 9) and adding surface area constraint and volume exclusion enhances the shape integrity, we conclude that the interaction between these elements benefits the overall dynamics. Thus, our model hints at a feedback mechanism between the membrane and the skeleton that goes beyond the accepted function of the spectrin skeleton in passively giving mechanical support to the membrane (Li, et al., 2023).”

R2.13) Fig.7F shows that changes are still significant at the final times, so these are not at steady-state yet.
Figure 7F (now 6F) shows the number of myosin edges over time. Since there is stochastic addition

© 2025. Published by The Company of Biologists under the terms of the Creative Commons Attribution License (https://creativecommons.org/licenses/by/4.0/).

and removal of spectrin edges, there are changes in the numbers at any time. Note that the changes in numbers in the case of area constraint and volume exclusion (yellow line) have minimal changes after 200 seconds (the difference is +- 1 myosin edge). Therefore, we consider this to be a minimal change. The other cases (simple case in blue and only membrane area constraint in red) have more drastic changes in the number of myosin edges.

R2.14) Page 19, it says:"J). These correlative observations closely resemble the series of events captured by our model, suggesting that the enhanced stability of the adherent meshwork is important for cell function." Its not clear what observations exactly correspond to what the model predicts, ie which results in panels A-F correspond to the experimental observations.
Why dont they plot the cell adhered area and height as in the experiment for comparison ? The claimed agreement is not clearly shown.
We appreciate the suggestion of the reviewer and have included the requested plots. Moreover, we have edited the text to include these observations (lines 367-368): "Our modeled adhered cell considering membrane area constraint and volume exclusion shows a hindered increase in projected cell area (Fig.6L) and it flattens over time (Fig. 6M)."

R2.15) Fig.8: Values and units are missing along the y-axis in H,J.
The values have been added.

R2.16) Page 21: "as on a recent model"->as in a recent model
Done.

R2.17) Table 1: the bending modulus of (400kBT) is much larger than values measured in previous studies (see refs in (1)).
We thank the reviewer for this observation. Indeed, there is a typo in our table, it should read 820 [pN nm] = **200** k_BT, we are taking k_BT = 4.1 [pN nm]. This is the value used in Li, et al., 2005, which we followed for the membrane bending. We recognized that this value is larger than the commonly used values but it allows stabilization of the concave shape of RBC in Li, et al., 2005, and results in better simulations in our model. We have added rationale for our choice of bending modulus (lines 478-482): "When we introduced the area constraint in the suspended cell, we took the sphere as the permanent reference state (i.e., A_0 is the initial area of the sphere) as in (Li, et. al, 2005). Thus, we used their proposed value of k_b=200k_BT, which is larger than the commonly used values of k_b but results in more stable shapes.".

Reviewer 3:

SUMMARY OF THE ADVANCE MADE IN THIS PAPER AND ITS POTENTIAL SIGNIFICANCE TO THE FIELD
This manuscript develops a computational biomechanics model for the deformations of a cell's actin-spectrin skeleton coupled to the cell membrane. The model incorporates key mechanical contributions to this cellular system: the bending elasticity of the membrane, surface and volume constraints from the membrane and cytoplasm, as well as the turnover of myosin motors and spectrin structures, and shows that these factors contribute to the stability of cell shape. While the modeling is thorough and can potentially inform future experiments on this important biophysical system, I have reservations for publication in current form detailed below. These mainly center on some modeling choices that are not made clear, and the lack of any quantitative comparison with experiments (or even a discussion of what testable predictions the model makes that moves understanding of cell biological mechanisms forward).
We thank the reviewer for their thoughtful feedback. We have addressed all of the reviewer comments. Because our model aims to give a general description of the membrane skeleton without considering the cell type, a quantitative comparison with experiments is hard as different cell types have different properties. However, in our manuscript, we observed different mechanisms of the membrane skeleton that promote shape transitions and shape stability and proposed different experiments. For example, in the discussion (lines 419-420), we suggested: "Experiments could test whether increasing or decreasing the number of myosin rods acting on the actin-spectrin meshwork enhances its response after shear stress is induced.". Another example is in lines 452-453: "The predictions derived from our model can be tested experimentally. For example, the optimum number of myosins acting on the actin-spectrin meshwork to promote its

© 2025. Published by The Company of Biologists under the terms of the Creative Commons Attribution License (https://creativecommons.org/licenses/by/4.0/).

recovery after the imposed stresses are removed and the enhanced stability of the adherent cell in comparison with the suspended cell.”. We hope that future efforts will provide a better merger of direct experiments and modeling.

SUGGESTIONS TO AUTHORS

Major

R3.1. Erythrocytes (red blood cells) are the prototypical cells that contain a 2D polygonal lattice structure of actin and spectrin. This seems to inspire the geometry of the spring network used in this model. It is not made clear if the model is specific to erythrocytes, or do the authors have other cells in mind as well? In the former case, the abstract and Discussion should specifically mention red blood cells. Some comments on how the model can be generalized to spectrin-actin skeleton in neurons and fibroblasts would also broaden the scope of the work.
This model is not specific to erythrocytes, we have now clarified this in the introduction by adding the following sentence (lines 95-96): “Because this interaction between spectrin and myosin is found in many types of cells (Xu, et al., 2013, Costa, et al., 2020, Ghisleni, et al., 2020, Ghisleni, et al., 2024, Nowak, et al., 2022), our model aims to give a general description and its findings are not exclusive to a certain cell type.”
We have added the following sentence to the discussion section to point out that our model is not cell-specific and how it can be modified to quantitatively match experimental data (lines 387-390): “Our model focuses on the mechanical properties of the spectrin skeleton, which we assumed to be shared between different types of cells, and is based on experimental observations in RBC and fibroblasts. Thus, our comparisons with experimental data are only qualitative. A quantitative comparison would require taking the parameters from experimental data for a specific cell type and perhaps adding cell-specific elements to the model.”
To avoid confusion about our general spectrin membrane skeleton model with a RBC model, we have specified that our initial mesh choice has reasons beyond resembling the RBC by modifying the following sentence in the Results section (lines 210-211): “**Although this configuration resembles the RBC membrane skeleton configuration, we chose it because** it allowed us to have a steady-state initial configuration (i.e., all spectrin edges have the same length) and quantify the deformations induced by mechanical stresses.”

R3.2. As mentioned in the Discussion, other modeling approaches describe spectrin as worm like chains. What justifies the neglect of bending and buckling of the spectrin? Maybe the authors can provide some estimate of the persistence length/bending elasticity of spectrin that justifies this choice. Further, it is well known that bending stiffness has an important stabilizing effect on disordered elastic networks (see for example, Broedersz et al. Nat. Phys. 7, 983-988 (2011); Das et al. PLOS ONE 7, e35939 (2012)) This effect may be important if the authors go away from their fully connected polygonal lattice geometry, and this regime may also occur in the current model when spectrin springs are removed. Have they measured the mean connectivity (coordination number) of their network in time? Do the simulated networks remains above the critical coordination number given by Maxwell's count?
We chose to model spectrin as simple Hookean springs because we aimed to study the interaction between different forces in the whole cell and this representation facilitated simulations. We had to make some design decisions to facilitate the simulations at the length scale of the whole cell. As pointed out in the discussion, the simulated spectrin extensions are within the small extension criteria (Svetina, et al. 2016). Thus, we assumed that our Hookean springs generate the same potential as the WLC model.
We agree with the reviewer that the bending elasticity of filaments modeled as WLC could have an effect on the network and have added this observation to the discussion (lines 441-442): “Bundles in WLC models can bend, and thus, networks using WLC description for fiber edges have given insight into the effect of single fiber bending rigidity on the network (Broedersz, et al. 2011).” However, simulating spectrin as a WLC model is out of the scope of our manuscript. The Maxwell’s count (z) in 3D is 6. In a network with N nodes in d dimensions subject to N_C constraints in the form of edges connecting the nodes, the average coordination number is $z = 2N_C/N$ (Wyart et al., 2008). Thus, Maxwell’s count denotes when the number of degrees of freedom and constraints is balanced ($Nd = N_C$), resulting in an isostatic (just rigid) system (Wyart et al., 2008). By design, our initial mesh is hexagonal, and thus, z is close to 6 (z=6-δ, δ<<1). We remove

© 2025. Published by The Company of Biologists under the terms of the Creative Commons Attribution License (https://creativecommons.org/licenses/by/4.0/).

spectrin edges and only add the spectrin edges that were previously removed (connecting the same nodes). Therefore, the network cannot go beyond the critical coordination number (i.e., z remains less or equal to 6).

R3.3. What is the experimental evidence that justifies the removal criterion of spectrin edges under compression? This should be discussed at the outset. Also, this choice has an interesting analogy with models that include buckling of fibers, since a buckled bond provides negligible resistance to compression.
In Module 1, section Spectrin unbinding and rebinding (line 109), we provided the evidence for spectrin removal "Spectrin dissociates the dimer-dimer links by proteolytic cleavage (An, et al, 2002)."
We mentioned in more detail this experimental evidence in Results, Unbinding of spectrin edges lower stresses due to shear deformation section (lines 245-246): "However, experimental evidence shows that the spectrin network in RBC can deform and experience high shear stress (An, et al, 2002). To do so, spectrin tetramers dissociate to dimers when a low shearing force is applied (An, et al, 2002)"

R3.4. It is known that myosin binding to actin involves catch bonds, i.e. a force dependent on and off rate. Can the authors include this in their model to see how the results change?
In this paper, we only model the effect of myosin dipoles acting on the spectrin meshwork, and the short actin filaments were considered as connection points of spectrin bundles. In the revised manuscript, we recognized that myosin dipole action can lead to aster formation, which can eventually stabilize into stress fibers by the action of catch bonds. We have added the following text to the Discussion section (lines 425-418): "Although theoretical models have studied the aster formation and stabilization to stress fibers due to myosin action (Vernerey, et al.,2016), this consideration is out of scope for our model because short actin filaments are represented as nodes. Thus, our model only considers a pulsative action of myosin, as reported in (Ghisleni, et al., 2023).".

R3.5. Fig. 3 B shows that the membrane is still high when membrane bending is included. The plot in 3B for meshwork height with bending included also shows highly blue and red regions that have high positive and negative heights. This is apparently contradictory to the authors claim in the text that bending reduces height fluctuations . The claim is not supported by the plot in 3E. The difference between the box plot for the case of compression and compression with bending are not apparent.
The reviewer is right, the meshwork with compression including bending shows highly blue and red regions. In our claim that bending energy reduces height fluctuations, we focused on the differences between nearby zones and not on the maximum and minimum height. To better show that the case with bending conserves the height differences lower within nearby regions we have added a supplementary plot (Fig. S2). We calculated the height difference between the endpoints of the spectrin edges. We found that the medians of the compressed cases with and without bending are significantly different (two-sided Wilcoxon rank sum test with p-value = 0.384). We have modified the text to include the reference to this supplementary Figure (line 228): "Moreover, adjacent nodes have smaller height differences (Fig. S2)."

R3.6. What observations in the cell spreading experiment can be quantitatively explained by this model? Can the model be used to generate cell area vs time plots like those shown in Figs. 8H and 8J? What are the testable predictions of the model that may be checked in future experiments?
We have added these plots to Figure 8 (now Figure 7, see response R2.14)

R3.7. Can the model say anything about the contributions of the spectrin skeleton to the old problem of flickering of the membrane of RBCs? An important reference on this topic: Turlier et al. Nature Phys 12, 513-519 (2016).
See our response to reviewer 2, R2.3, where we commented on the flickering of the RBC membrane.

Minor
R3.minor.1. Line 261, the figure referenced should be 2A and not 2B.
Thank you. Done.

© 2025. Published by The Company of Biologists under the terms of the Creative Commons Attribution License (https://creativecommons.org/licenses/by/4.0/).

R3.minor.2. Line 288: F-actin z-node position may have some extra points inside the box plot for the case of compression with bending but it also has a higher range in both positive and negative side - while the median is about the same. We suggest that the actual values of the median be reported and their difference be checked for statistical significance.
See our response to R3.5.

R3.minor.3. Fig. 4G has two green colored dashed lines. What is the difference between them?
We think there is a misunderstanding - the top lines in the plot correspond to the blue dashed line and the yellow dashed line. Because they are very close to each other it can be perceived as a green line. We have changed the yellow line to a marker to avoid confusion.

R3.minor.4. Fig 4D: When meshwork is released (in both unbinding and unbinding+rebinding cases), it should have equal amounts of tensile and compressive forces - as mandated by force balance. However, from plots in A, it seems that most of the spectrin edges are compressed and apply a positive force. This deviation from rest-length is however pretty small as per 4D. It would be more instructive to apply a threshold for the color of edges so that the edges appear to be at rest-length then or an explain the deviation from rest-length.
In our selected color scheme for the force in 4A (now Figure 3), pink corresponds to 0 force. Compare the initial configuration at the left plot of 4A, where the spectrin edges equal the resting length (180 nm) to the plots for released unbinding and unbinding+rebinding cases at the right. Both have the same color for the spectrin edges. To be more precise, we have included the values for the median in 4D and a Wilcoxon signed rank test that fails to reject the null hypothesis that the median of the length minus the resting length is equal to zero with p-value of 0.0773 for the Final ub released distribution and 0.9582 for the Final ub rb released distribution.

R3.minor.5. In Fig. 4D, a horizontal line at y=180 nm would be useful.
We have included the mean values for the boxplot (see R3.minor.4). We think those values are more informative than the suggested line.

R3.minor.6. Line 333: In the model, spectrin rebinding happens when the distance between two F-actin nodes becomes equal to rest-length. In practice, does this mean that there is a small threshold around the 180 nm rest length at which the spring reappears?
We thank the reviewer for this excellent observation. Yes, in practice the actin nodes could move beyond 180 nm in a time step and therefore, there is a small threshold around 180nm. We have changed "... was equal to the resting length" to "... was equal to or larger than the resting length."

R3.minor.7. Fig. 6C: the percentage of attached edges seems to be slightly above the value for rr/5 and 5rr. Although two values greater than rr have been used to establish this convincingly (5rr and 10rr), it may make this work better with another value smaller than rr is used as well apart from rr/5. Moreover, since 30 simulations have been used, how statistically significant is the difference between rr and rr/5?
Preliminary simulations using the value rr/10 resulted in either no addition or fast removal of myosin edges. Hence, over time, the number of myosin edges tended to zero. We thought this case was not relevant for describing the effect of myosin addition and removal rates in the meshwork, and therefore, we did not include it in the results. Note that the mean number of myosin edges in the simulation with rr/5 (Fig. 6A, now 5A) is already close to zero. There is no significant difference in the mean percentage of attached edges at the end of the simulation between rr and rr/5. However, the difference between the mean percentage of attached edges at the time when the network is released and at the end time is higher for rr/5 (3.8048%) than for rr (3.2809%).

R3.minor.8. 6E: the box plot of spectrin edge lengths does not convincingly establish why rr is a better parameter than other values, specifically, rr/5. 6F: it is also not abundantly clear form the height data why rr is a better parameter than rr/5, as claimed in line 387. Lines 383-385 should also mention the corresponding IQR values for rr/5 and establish how this is significantly worse than those for corresponding values of rr.
We agree with the reviewer that the IQR values for rr/5 do not clearly differ from the IQR of rr. However, our claim that "we concluded that our original parameters, which resulted in a single myosin per 2.5 μm2 acting on the meshwork after releasing it from focal adhesions, gave a more

© 2025. Published by The Company of Biologists under the terms of the Creative Commons Attribution License (https://creativecommons.org/licenses/by/4.0/).

efficient recovery." also refers to the difference between the mean percentage of attached edges at the time when the network is released and at the end time (see R3.minor.7). We have made this clarification by adding the following sentences (lines 302-305): "When decreasing the parameters to rr/5, the IQR for spectrin length (1.6163), actin node height (0.9195), and membrane force magnitude (0.0150) were similar to the original value. However, the difference between the mean percentage of attached edges at the time when the meshwork is released and at the end time is higher for rr/5 (3.8048%) than for rr (3.2809%). Thus, the meshwork with a smaller rate ratio has a slightly worse recovery."

R3.minor.9. Line 421: Myosin removal is a purely stochastic event so why should the change in force balance lead to the subsequent removal of myosin?
The myosin edges are also removed when their length is below a threshold d_{min} (see Module 4: Myosin dynamics). In previous line 421: "We also observed that the number of myosin edges was higher (Fig. 7F), which resulted from the change in the force balance that hindered the myosin contraction, and thereby, their removal.", we referred to the addition of the area constraint that changed the force balance. Thus, the dynamics of the actin nodes are now dictated by the balance between the spectrin spring force, the myosin contraction, the membrane bending, and the membrane surface area constraint. The effect of the area constraint is to counteract the contractile force generated by the myosin edges, slowing down the change in position of the actin nodes and thus, the location of the ends of myosin edges. This slows down the contraction of the myosin edges and delays their removal when they are shorter than d_{min} .

R3.minor.10. Fig. 3B: Why does addition of bending membrane force break the left-right and top-bottom symmetry when compared with the case of isotropic compression without bending? Even with bending force, the symmetry should be maintained.
We used Eq. 3 to calculate the bending force which minimizes the angle formed by two adjacent triangular faces of the mesh (Fig 2B, now S2B). We believe that this local definition of bending leads to the geometry breaking.

R3.minor.11. Fig. 5G: There does not seem to be a significant difference between the two cases without myosin (dashed lines) and with myosin (solid lines). This does not support the statement at line 356.
In line 356, we wrote "myosin increased the rebinding events after the meshwork was released", which now we have demonstrated that there is a significant difference. See response R2.8.

REFERENCES

Ghisleni, A., Bonilla-Quintana, M., Crestani, M., Lavagnino, Z., Galli, C., Rangamani, P. and Gauthier, N.C., 2024. Mechanically induced topological transition of spectrin regulates its distribution in the mammalian cell cortex. Nature Communications, 15(1), p.5711.

Li, J., Dao, M., Lim, C.T. and Suresh, S., 2005. Spectrin-level modeling of the cytoskeleton and optical tweezers stretching of the erythrocyte. Biophysical journal, 88(5), pp.3707-3719.

Wyart, M., Liang, H., Kabla, A. and Mahadevan, L., 2008. Elasticity of floppy and stiff random networks. Physical review letters, 101(21), p.215501.

Vernerey, F.J. and Akalp, U., 2016. Role of catch bonds in actomyosin mechanics and cell mechanosensitivity. Physical review e, 94(1), p.012403.

<u>Second decision letter</u>

MS ID#: jcs.263473

MS TITLE: Dynamic mechanisms for membrane skeleton transitions

© 2025. Published by The Company of Biologists under the terms of the Creative Commons Attribution License (https://creativecommons.org/licenses/by/4.0/).

AUTHORS: Mayte Bonilla-Quintana; Andrea Ghisleni; Nils C. Gauthier; Padmini Rangamani
ARTICLE TYPE: Research Article

I am happy to tell you that your manuscript has been accepted for publication in Journal of Cell Science, pending standard publication integrity checks.

© 2025. Published by The Company of Biologists under the terms of the Creative Commons Attribution License (https://creativecommons.org/licenses/by/4.0/).
